# Metabolic changes and biochemical degradation during dark anoxic incubation of *Nannochloropsis*: implications for low-energy microalgal cell rupture

**DOI:** 10.1007/s00449-025-03185-7

**Published:** 2025-06-09

**Authors:** Bhagya Yatipanthalawa, Esther Mienis, Ronald Halim, Imogen Foubert, Muthupandian Ashokkumar, Peter J. Scales, Gregory J. O. Martin

**Affiliations:** 1 Algal Processing Group, Department of Chemical Engineering, The University of Melbourne, Parkville, VIC 3010 Australia; 2 Research Unit Food & Lipids, Department of Microbial and Molecular Systems (M2S), KU Leuven Kulak, E. Sabbelaan 53, 8500 Kortrijk, Belgium; 3 UCD Algae Group, School of Biosystems and Food Engineering, University College Dublin, Belfield, Dublin 4, Ireland; 4 Sonochemistry Group, School of Chemistry, The University of Melbourne, Parkville, Melbourne, VIC 3010 Australia

**Keywords:** Microalgae, Proteolysis, Lipolysis, Dark anoxia, Cell rupture

## Abstract

Dark anoxic incubation has been identified as a low-cost method to facilitate the mechanical rupture of microalgae such as *Nannochloropsis* via autolysis-induced cell wall thinning. During this process, concentrated slurries of cells are incubated in the dark at an elevated temperature, to deprive them of light and oxygen. This work analyzed the integrity of proteins and lipids during dark anoxic incubation and investigated the cellular responses of *Nannochloropsis* through an in-depth proteomic analysis. Proteomic analysis identified enzymes associated with cellulose hydrolysis and glycolytic and fermentative pathways that are presumably activated to produce energy in the absence of light and oxygen. Progressive biochemical degradation was observed during 48 h of incubation, including the proteolysis and leakage of proteins, and the lipolysis and subsequent peroxidation of lipids. This provides further evidence of autolytic processes occurring during prolonged incubation, which can be attributed to uncontrolled action of intracellular proteases and lipases. Importantly, the resultant formation of peptides and free fatty acids will affect their use in food and fuel applications. It is therefore important to optimise the incubation time and parameters to achieve cell weakening while minimising the unnecessary degradation of biomacromolecules**.**

**Graphical abstract:**

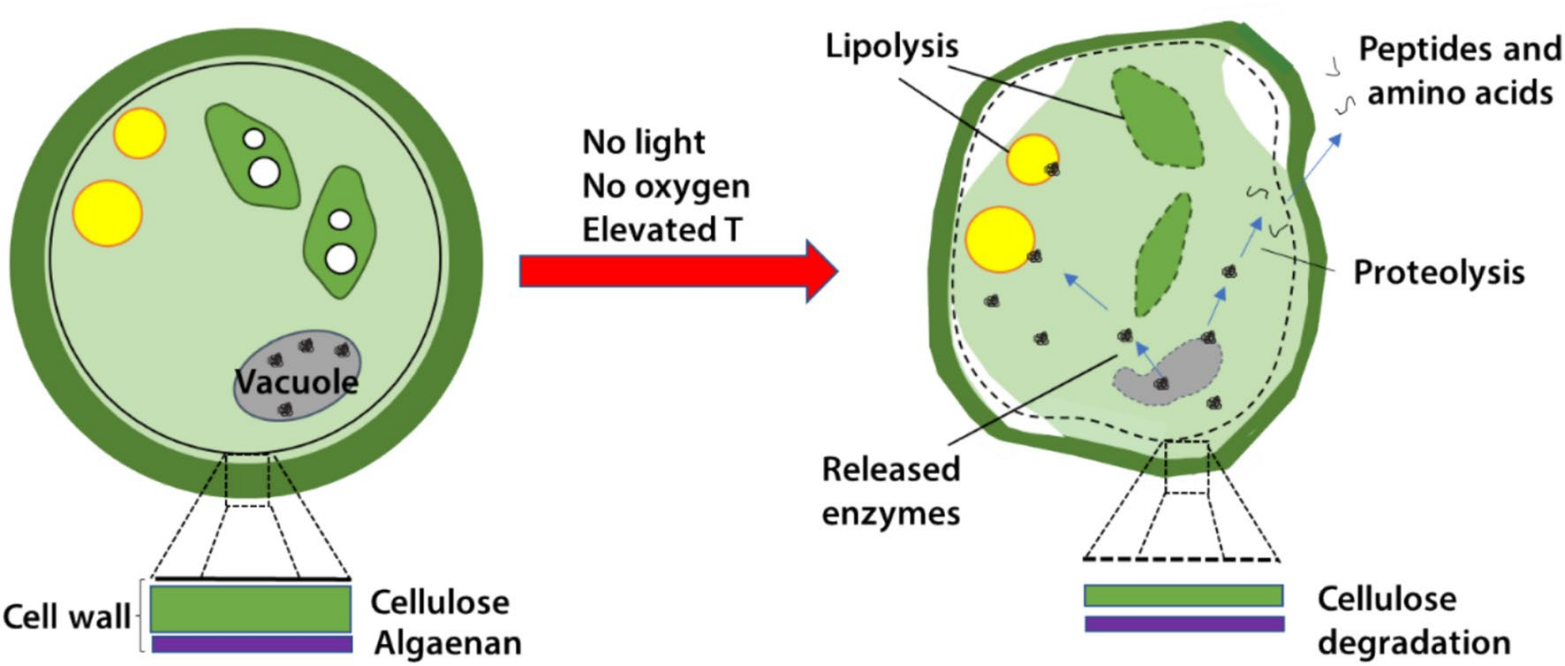

**Supplementary Information:**

The online version contains supplementary material available at 10.1007/s00449-025-03185-7.

## Introduction

With the increasing demand for sustainable resources, microalgae are increasingly being researched as a renewable feedstock for food and fuel [1, 2]. Microalgae contain lipids, carbohydrates, proteins and can produce high-value metabolites such as long chain polyunsaturated fatty acids, carotenoids and vitamins [2]. Due to the presence of robust cell walls, the extraction of valuable components often requires cell rupture. Amongst the diverse range of microalgae, cell walls are often composed of polysaccharides such as cellulose, xylan, mannose and other components such as uronic acids, proteins, glycoproteins, algaenan and sometimes even silica [3, 4].

*Nannochloropsis* sp., a promising precursor for algae-based lipid production due to its high TAG accumulating ability, have particularly strong cell walls that are resistant to cell rupture [5]. A low-cost method was introduced previously to weaken these strong cell walls, making the subsequent physical rupture easier [6, 7]. Here concentrated slurries of cells were subjected to dark anoxic incubation (deprivation of light and oxygen) at an elevated temperature, which led to self-digestion of the cell walls [8]. Despite long residence times (up to 24 h), at high-solids concentrations the volume of the incubation vessel is minimised. As a simple holding tank requiring only minimal mixing and insulation, the vessel is very low-cost to construct and operate. Further, the heat required to bring the contents up to the required temperature (35–40 °C) can be recovered from down-stream operations.

When photosynthetic microalgae are deprived of light, they cease photosynthetic oxygen production, and instead consume oxygen for respiration drawing on internal carbon reserves [9]. This can eventually lead to anoxic conditions. Oxygen is essential for energy production through oxidative phosphorylation, and certain biosynthetic pathways requiring oxygen as an oxidant or a reagent [10]. A lack of oxygen therefore requires adjustment of cellular pathways through differential expression of genes. Previous studies have found microalgae such as *Chlamydomonas* have the necessary enzymes and pathways to survive dark anoxic conditions. To generate the energy needed for survival, they ferment plastidic starch into a variety of end products such as acetate, ethanol, formate, glycerol, lactate, H_2_ and CO_2_. Some microalgae excrete the fermentation products while others accumulate them inside the cells [11, 12]. For example, *Chlamydomonas* was found to excrete different fermentation products such as ethanol, malic acid, acetic acid and formic acid into the surrounding medium [12], whereas *Euglena gracilis* undertakes wax ester fermentation, during which the wax ester products are retained within the cells [13].

Our previous study showed that subjecting *Nannochloropsis* sp., to dark anoxic incubation led to the formation of fermentative end products, and concomitant depletion of carbohydrate reserves, suggesting they employ fermentative pathways similar to other studied microalgae species [8]. The utilisation of intracellular carbohydrate reserves resulted in the depletion of cell-wall cellulose which rendered the biomass more susceptible to subsequent disruption and improved biorefinery performance. The study was the first to investigate the acclimation of *Nannochloropsis* to dark-anoxic incubation and associated cell-wall degradation. However, the exact biochemical pathways used by *Nannochloropsis* sp. to respond to dark anoxic stress conditions were not investigated in the study.

Much is already known about the metabolic responses of the well-characterised and studied microalgae *Chlamydomonas*. Less is typically known about other microalgae, but it is clear that the metabolic responses of different microalgae species under stress conditions can differ. For example, in a previous study, the diatom *T. weissoflogii* could survive under darkness, whereas 6 days of darkness resulted in cell death of the chlorophyte *D. tertiolecta* [14]. While some effects of dark anoxic incubation on the cell physiology of *Nannochloropsis* have been revealed in our previous studies [8, 15], it is of interest to understand the cellular responses in more detail, which can then be compared with other species. *Nannochloropsis* sp. is a marine alga that dwells in marine environments, which can frequently become anoxic. An in-depth understanding of the cellular response of *Nannochloropsis* sp. to dark-anoxic incubation is relevant to understanding the behaviour and decomposition of this microalgae in the marine environment. In addition, this knowledge is needed to understand the properties of microalgal biomass during wet storage (where conditions would likely result in dark anoxia) and inform appropriate preservation strategies to stabilise the biomass and its composition. For biorefinery fractionation of the biomass, the insights gained from understanding cell response to dark anoxia can be used to optimise incubation pre-treatment to facilitate efficient recovery of high-value components without compromising the quality of the products.

Halim, et al. [8] previously observed that the total lipid and protein content of *Nannochloropsis* sp. was not significantly impacted by dark anoxic incubation. However, no measurements were performed to investigate potential biochemical alterations to the proteins or lipids. Proteins are of practical interest as a product from microalgae. The compositional profile will influence the nutritional and functional value of proteins. Further, the quality of the proteins could be affected by the incubation process. It is possible that proteolysis may occur, which could have several practical implications. Some peptides could be bioactive [16, 17], while others could be bitter [18] or have altered biophysical functionality [19]. In addition, the release of peptides from the cells could affect protein yields.

Lipids are another potential commercial product from microalgae. Lipid hydrolysis and lipid peroxidation can reduce lipid quality, and these processes have been observed in plants during anoxic conditions [20]. Previously, Balduyck, et al. [21] also found that the free fatty acids (FFA) content in *Nannochloropsis* sp. increased during wet storage at 4 °C and 20 °C. The storage conditions were both dark and presumably anoxic, while the temperature was lower and the incubation period longer than that used for cell weakening (< 2 d). It was seen that the polar lipids were mostly affected, and the activity of a phospholipase was proposed. Such degradation of the lipids can have implications in different applications, such as downstream processing problems in biodiesel production and rancid flavours and loss of nutritional value for food applications [22].

Microalgal cells contain lipases, proteases and cellulases of which the activity is systematically controlled by and compartmentalised within the cells. It is possible that dark anoxic incubation could result in active overexpression of these enzymes or decompartmentalization/intracellular release resulting in inadvertent degradation of cellular proteins, lipids and cell wall material.

The current study aimed to identify the cellular response to dark anoxic incubation using a combined proteomic and biochemical analysis. Specifically, the study investigates differences in the proteome when subjected to dark anoxic incubation, in particular the expression of proteins governing fermentative energy generation during dark anoxia, and the occurrence of proteolysis and lipolysis. The effects on the overall biochemistry of the cells are also investigated, focussing on the practical implications of *Nannochloropsis* dark-anoxia acclimation on the use of incubation pre-treatment for biorefinery processing.

## Materials and methods

### Microalgae strain and cultivation

A previously studied microalgae *Nannochloropsis salina* was grown indoors at 20 °C in multiple 12 L carboys in parallel with a light:dark cycle of 14:10 h [7]. A modified f/2 medium [23] was used and each bioreactor was aerated with an aquarium air pump (Stellar 380D, Aqua One, China) at a flow rate of 190 L/h, which was split into two air stones. Algae were harvested using a disc stack centrifuge (Separator OTC 2–02-137, GEA Westfalia, Italy) at the end of a 14-day growth cycle.

### Dark anoxia incubation

Three separate batches of freshly harvested microalgal biomass cultivated under identical conditions were used in independent investigations into the effect of dark anoxic incubation on (i) the release of proteins and peptides, (ii) lipid hydrolysis and oxidation, and (iii) proteomic profile and relative proteolysis (RP).

Algae paste was diluted in synthetic seawater medium (30 g/L Red Sea Coral Pro salt dissolved in deionised water) to a salt-free dry weight solids concentration of ~ 12% w/w. For each experimental replicate, 80 mL of algae slurry was put into 100-mL Schott bottles and covered in aluminium foil to create dark conditions. Two holes in the lid were used to allow entry and exit of nitrogen gas which was sparged for 30 s prior to incubation to ensure anoxic conditions. For incubation, the bottles were kept in a water bath maintained at 38 ± 1 °C. Stirring was not provided during incubation as previously mixing was shown to have no impact on incubation except for maintaining a uniform temperature in a large vessel [8]. In the small vessels used in these experiments a consistent temperature was maintained using a hot plate stirrer (Stuart CC162, Crown Scientific, NSW). Duplicate 7.5-mL samples of algae slurry were taken from each vessel after 8 h, 24 h, and 48 h of incubation for analysis. Before sampling, the contents in the vessel were hand mixed and pipetted up and down several times to ensure homogeneity of the sample. Following sampling, containers were sparged again with nitrogen as mentioned above and the incubation was continued.

### Protein and peptide release during incubation

Samples taken from the incubation vessels were centrifuged at 9000 g for 10 min (Allegra X-30R, fixed-angle rotor F0685, Beckman Coulter, Victoria, Australia). The supernatant was carefully recovered without disturbing the algae pellet. The total protein in the supernatant fraction was quantified using a modified Lowry protein assay kit (Pierce^™^ Modified Lowry Protein Assay kit, Thermo scientific) following appropriate dilutions using synthetic seawater medium. Peptides in the supernatant fraction were separated from whole proteins using a 30-kDa membrane filter (Millipore, Merck) and centrifuged at 10,000 g for 10 min using an Eppendorf 5702 fitted with an A-4–38 rotor (Eppendorf, North Ryde, NSW, Australia) to obtain the peptide-rich fraction in the supernatant. The total peptide content was measured using the modified Lowry assay.

### Cell rupture and protein extraction

To assist in the complete recovery of proteins for total protein quantification, RP measurement and proteomic analysis, cells were first ruptured by high-pressure homogenisation. Samples of algae from the incubation experiments and the unincubated/fresh algae were diluted with deionised water ten times to obtain a sufficient volume to be processed in the high-pressure homogeniser (Panda 2 K NS1001L, GEA Niro Soavi, Parma, Italy) and a protease inhibitor (Sigmafast protease inhibitor, Sigma-Aldrich) was added to halt any proteolysis. The algae slurry was passed through the homogeniser 5 times at a pressure of 1000 ± 100 bar. Following cell disruption, 35 mL of the algae slurry was put into a 50-mL centrifuge tube (Falcon) and centrifuged at 4000 g for 10 min (Allegra X-30R, fixed-angle rotor F0685, Beckman Coulter, Victoria, Australia). Supernatants were collected and the pellets redispersed in 10 mL of 2.5% SDS, 50 mM Tris pH 8.1 buffer to maximize the protein extraction for the subsequent proteomic studies. Samples were vortexed for 1 min, following which they were centrifuged again at 4000 g for 10 min to remove cell debris. Supernatants from the repeated centrifugations were pooled together and used for subsequent quantification of the total protein content, RP measurements and proteomics.

### Relative proteolysis

An assay was performed on the incubated samples to estimate the extent of proteolysis relative to the unincubated cells (sample at *t* = 0 h). This was done by measuring the number of free amino groups present in the supernatants described above, using the O-phthalaldehyde (OPA) reagent. OPA reagent was prepared as previously described [24]. In brief, 10 mL of 50-mM OPA solution in methanol, 10 mL of 50 mM N-acetyl cysteine (NAC) in water and 5 mL of 20% w/v sodium dodecyl sulphate (SDS) solution were dissolved in 75 mL of 0.1 M borate buffer (pH 9.5). Twenty µL of the diluted supernatant fraction was mixed with 2.4 mL of the reagent and the absorbance was read at 340 nm after incubating for 2 min. The background noise of the supernatant was measured at 340 nm after diluting 20 µL of the diluted supernatant fraction in 2.4 mL of deionised water and subtracted as a baseline. A standard calibration curve of leucine was used to represent free amino acids. The % RP of the protein fractions of the incubated samples was then calculated using the following equation:

$$R{P}_{x}=\left[\left(\frac{\Delta Abs 340nm of x h incubation}{\Delta Abs 340nm of 0 h incubation}\times \frac{Average protein content at t=0h}{Average protein content at t=xh}\right)-1\right]\times 100$$

where $$x=8h,24h,48h$$

Samples were measured in duplicate for triplicated experiments. The average protein content was measured using a BCA assay kit (Micro BCA^™^ Protein assay kit, Thermo Scientific) using the microplate procedure according to the supplier’s procedure.

### Protein sample preparation for LC/MS/MS

Protein samples obtained following cell rupture, were purified for proteomic study using a previously described procedure [25]. Based on the protein content measured using the BCA assay, an appropriate volume was pipetted into a 2-mL microfuge tube to obtain a protein mass of around 0.1 mg. To precipitate the proteins ice-cold acetone was added (at 4 × the volume of the pipetted sample) and the tubes left in the freezer at -20 °C overnight. The tubes were then centrifuged at 16,000 g for 10 min using an Eppendorf 5702 fitted with an A-4–38 rotor (Eppendorf, North Ryde, NSW, Australia). The supernatant was removed, and the pellet was slowly washed with 1 mL of ice-cold acetone, recentrifuged, and the supernatant removed. The remaining pellet was resuspended in 100 µL of 8 M urea in 50 mM triethylammonium bicarbonate (TEAB) (Thermofisher Scientific) and vortexed to solubilise the pellet completely. The protein content was quantified to verify that a protein concentration of 1 ± 0.2 mg/mL was achieved.

Tris(2-carboxyethyl) phosphine (TCEP) was added to obtain a concentration of 10 mM in the sample (2 µL of 0.5 M TCEP) and mixed for 45 min at 37 °C. Iodoacetamide was added to reach a concentration of 55 mM (22 µL of 0.25 M iodoacetamide in 25-mM TEAB) and again mixed for 45 min at 37 °C. Finally, the solution was diluted to reach a final concentration of 1 M urea using 25 mM TEAB and was digested with trypsin (1:40) (Trypsin singles, Proteomics grade, Sigma-Aldrich, USA) shaking overnight at 37 °C using an incubated shaker (New Brunswick, Eppendorf, USA). Following trypsin digestion, solid phase extraction (SPE) was performed to purify the peptide fractions. The solution was acidified by adding pure formic acid (FA) to a concentration of 1% (v/v). SPE cartridges (Oasis HLB 1 cc Vac Cartridge, 10-mg Sorbent per Cartridge, Waters, Australia) were washed with 1 mL of 80% acetonitrile (ACN) containing 0.1% trifluoro acetic acid (TFA) (A), followed by two sequential washes with 1.2 mL of 0.1% TFA (B). The sample containing the peptide fractions was loaded into the SPE column and washed with 1.5 mL of B. The peptide fraction was eluted with 800 µL of solution A and collected in a 1 mL Eppendorf tube. Samples were dried in a SpeedVac concentrator (Labcono Centrivap Concentrator, Kansas, MO, USA) for 20 min to remove ACN and then were freeze dried overnight using a freeze dryer (Christ Alpha 1–2 LD Plus).

### Proteomic analysis

The freeze-dried peptide fractions were redispersed in 200 µL of the loading buffer (2% ACN and 0.05% TFA) and centrifuged at 16,000 g for 10 min (Eppendorf 5702 fitted with an A-4–38 rotor, Eppendorf, North Ryde, NSW, Australia) to remove any undispersed particles. Then the samples were subjected to mass spectrometric analysis as previously described [26]. In brief, an Orbitrap Elite mass spectrometer (Thermo Fisher Scientific) coupled online to a rapid separation liquid chromatography (RSLC) nano HPLC (Ultimate 3000 UHPLC, Thermo Fisher Scientific) was used. The nano HPLC column was equipped with an Acclaim Pepmap nano‐trap column (Dionex-C18, 100 Å, 75 μm × 2 cm) and an AcclaimPepmap analytical column (Dionex-C18, 100 Å, 75 μm × 50 cm). Peptide mix was injected to the enrichment column at an isocratic flow of 5µL/min of 3% (v/v) ACN containing 0.05% (v/v) TFA for 6 min, applied before the enrichment column was switched in-line with the analytical column. The eluents were 0.1% (v/v) FA in water (solvent A) and 100% (v/v) ACN in 0.1% (v/v) FA (solvent B). Flow gradient was (1) 0–6 min at 3% B; (2) 6–95 min 3 to 20% B; (3) 95–105 min, 20–40% B; (4) 105–110 min, 40–80% B; (5) 110–115 min, 80–80% B; (6) 115–117 min 85–3% and equilibrated at 3% B for 10 min before the next sample injection. The LTQ Orbitrap Elite spectrometer was operated in the data-dependent mode with nano-ESI spray voltage of 1.8 kV, capillary temperature of 250 °C, and S-lens RF value of 55%. All spectra were acquired in positive mode with full scan MS spectra from m/z 300–1650 in the FT mode at 240,000 resolution. Automated gain control was set to a target value of 10^–6^. Lock mass of 445.120025 was used. The top 20 most intense precursors were subjected to rapid collision-induced dissociation (rCID) with a normalized collision energy of 30 and activation q of 0.25. Dynamic exclusion of 30 s was applied for repeated precursors. Results were analysed using MaxQuant (v.1.6.2.3) against a UniProt database [27] for both *Nannochloropsis gaditana* and *Nannochloropsis salina*. The intensity based absolute quantification (iBAQ%) value was used for comparisons [28]. Identified proteins with a minimum of two peptides and a 1% false discovery rate (FDR) were included in the analysis. Data pre-processing was done using Perseus software. Three process replicates were used for the incubation time intervals. Sample groups were filtered to remove the proteins with a zero iBAQ intensity.

Proteins were filtered such that the iBAQ intensity was > 0 in at least 80% of the sample replicates in at least one time interval (0 h, 8 h, 24 h, 48 h). Values were then converted into log 2 values, which assigned “NAN” (i.e. Not a number as log₂0 = NAN) to the entries with an iBAQ intensity = 0. These values were imputed using the software guidelines to obtain a normal distribution. Following pre-processing, data were further manually analysed using Microsoft Excel. The relative abundance of the proteins was obtained using the iBAQ intensity following the equation below [29]:

$$riBAQ of protein P=\frac{iBAQ of protein P}{\sum_{i=1}^{n}iBA{Q}_{i}}$$

This gives an estimation of the protein concentration of the protein P in each individual sample. The average relative abundance of the protein P for the replicated samples were then obtained to compare between the incubation time intervals. The fold change was obtained by comparing the average relative abundance of the incubated samples for a given time t (t = 8 h, 24 h, or 48 h) with that of the fresh/unincubated sample (t = 0 h).

$$Fold change of protein P=\frac{Average riBAQ\left(t\right) of P}{Average riBAQ \left(0h\right)of P}$$

(t = 8 h, 24 h, 48 h).

The proteins were manually categorised into different protein groups based on their key cellular functions in *Nannochloropsis* sp using uniport database [27]. All these details are included in Supplementary 2.

### Lipid analysis

A separate batch of *Nannochloropsis* was prepared for lipid analysis using the same procedures described above, and subjected to dark anoxic incubation in three separate, replicate vessels, again following the same process discussed above. Samples taken during incubation were frozen at − 80 °C and then freeze dried. Freeze-dried samples were then stored at − 80 °C until being transported to KU Leuven Kulak, Kortrijk, Belgium for lipid analysis covered in Sect. Lipid extraction from microalgal biomass, Determination of the FFA content and Determination of the primary lipid peroxidation. The samples were transported in dry ice, via rapid air transport by Dangerous Goods International (DGI global). The integrity of the samples was confirmed by a comparison of the cell permeability using hexane isopropanol extractions done at both The University of Melbourne and KU Leuven (results not shown here).

### Lipid extraction from microalgal biomass

Total lipid content of the freeze-dried *Nannochloropsis* biomass was gravimetrically determined using chloroform/methanol (1:1 v/v) as described by Ryckebosch, et al. [30]. Briefly, 4 ml, 2 mL and 0.4 mL of methanol, chloroform and demineralised water respectively were added to 100 mg freeze-dried biomass and the sample was vortexed. 2 mL of both chloroform and demineralised water was added to the resulting sample, followed by vortexing and centrifugation. The upper phase was discarded, and the lower organic solvent phase was collected. The remaining biomass was re-extracted using 4 mL chloroform/methanol (1:1 v/v) followed by vortexing and centrifugation. The organic solvent phase was pooled with the previously collected organic solvent phase. The remaining biomass was subjected to a second extraction according to the same protocol and the organic solvent phases of both extractions were pooled. The collected organic solvent was filtered through a sodium sulphate layer. The total lipid content was gravimetrically determined after evaporation of the organic solvent.

### Determination of the FFA content

The total FFA content was determined by derivatisation of the FFA to ethylamine derivatives based on the method developed by Kangani, et al. [31] with slight modifications as described by Gheysen, et al. [32]. In brief, 10 mg of lipids obtained as described in the previous Sect. Lipid extraction from microalgal biomass were dissolved in 1 mL dichloromethane, following which a volume of 10 µL of diisopropylethylamine and a volume of 30 µL of diethylamine were added and the mixture was cooled to 0 °C. A 40 µL volume of bis(2-methoxyethyl) aminosulfur trifluoride was added dropwise into the mixture obtained, and the solution was vortexed for 5 s and kept at 0 °C for 5 min. Then the solutions were brought back to room temperature (25 min), 2 mL of demineralised water and 4 mL of hexane was added, and the solutions were mixed for 1 min. The solutions were centrifuged (750 g, 10 min) and the upper hexane layer containing the FFA derivatives were analysed using gas chromatography (GC) with cold on-column injection and flame ionization detection (FID) (Trace GC Ultra, Thermo Scientific, Interscience, Louvain-la-Neuve, Belgium) using a temperature profile as follows: 100–160 °C (20 °C/min), 160–240 °C (4 °C /min), 240 °C (27 min). The total FFA content was quantified by obtaining the sum of the peak areas and comparing it to the area of the internal standard used (C13:0).

### Determination of the primary lipid peroxidation

The quantification of primary lipid peroxidation was performed by determining the peroxide value (PV) using the spectrophotometric FOX method as described by [83]. 10 mg of lipids obtained as described in Sect. Lipid extraction from microalgal biomass, was dissolved in chloroform/methanol (7:3 v/v), diluted 1/10 and the absorbance (A_DS_) was measured at 560 nm. 50 µL of xylenol orange and 50 µL acidified Fe (II) chloride solution were added. The absorbance of the blank of the Fe(II) chloride solution (A_B_) and the absorbance of the sample solution (A_s_) at 560 nm was measured after exactly 5 min. Fe (III) chloride solution (10 µg/ml) was used to prepare a calibration curve of Fe (III) against its absorbance at 560 nm. The PV value was calculated according to:

$$PV=\frac{\left({A}_{s}-{A}_{DS}-{A}_{B}\right)* {m}_{i}}{W*55.84*2}$$

With m_i_ the inverse of the slope of the calibration curve, and W the mass of the lipid (g). The PV were normalised against the PV value of unincubated/fresh sample to obtain the change in peroxide value (ΔPV).

### Statistics and reproducibility

Incubation experiments were performed as triplicates, with three separate vessels used to incubate microalgae grown and harvested as a single batch. Subsequent protein extraction, RP measurement, lipid extraction, FFA measurement and lipid peroxidation value were all performed in duplicate for each of the triplicated experiments. The presented data points include the mean and standard deviation of all values (*n* = 6). For the unincubated/fresh sample, all the above measurements were performed in triplicates instead of duplicates. Spearman rank correlation analysis was conducted, treating each replicate value as an individual data point, to show the monotonic variation of these variables with incubation time, yielding *ρ* and *p* values [33]. The closer to 1 the absolute value of *ρ* is, the higher the correlation between two variables. A *p* value < 0.05 was considered statistically significant.

For the proteomic analysis, triplicated incubation experiments and duplicated protein extractions were performed. Individual protein samples were analysed, and the protein extraction duplicates were pooled together for each experiment when analyzing using the MaxQuant software. Statistical analysis was done on the individual proteins and the categorized protein groups. An ANOVA analysis was done followed by post-hoc t-tests (*p* < 0.05) to identify the significantly changed proteins and protein groups.

## Results

**Fig. 1:**
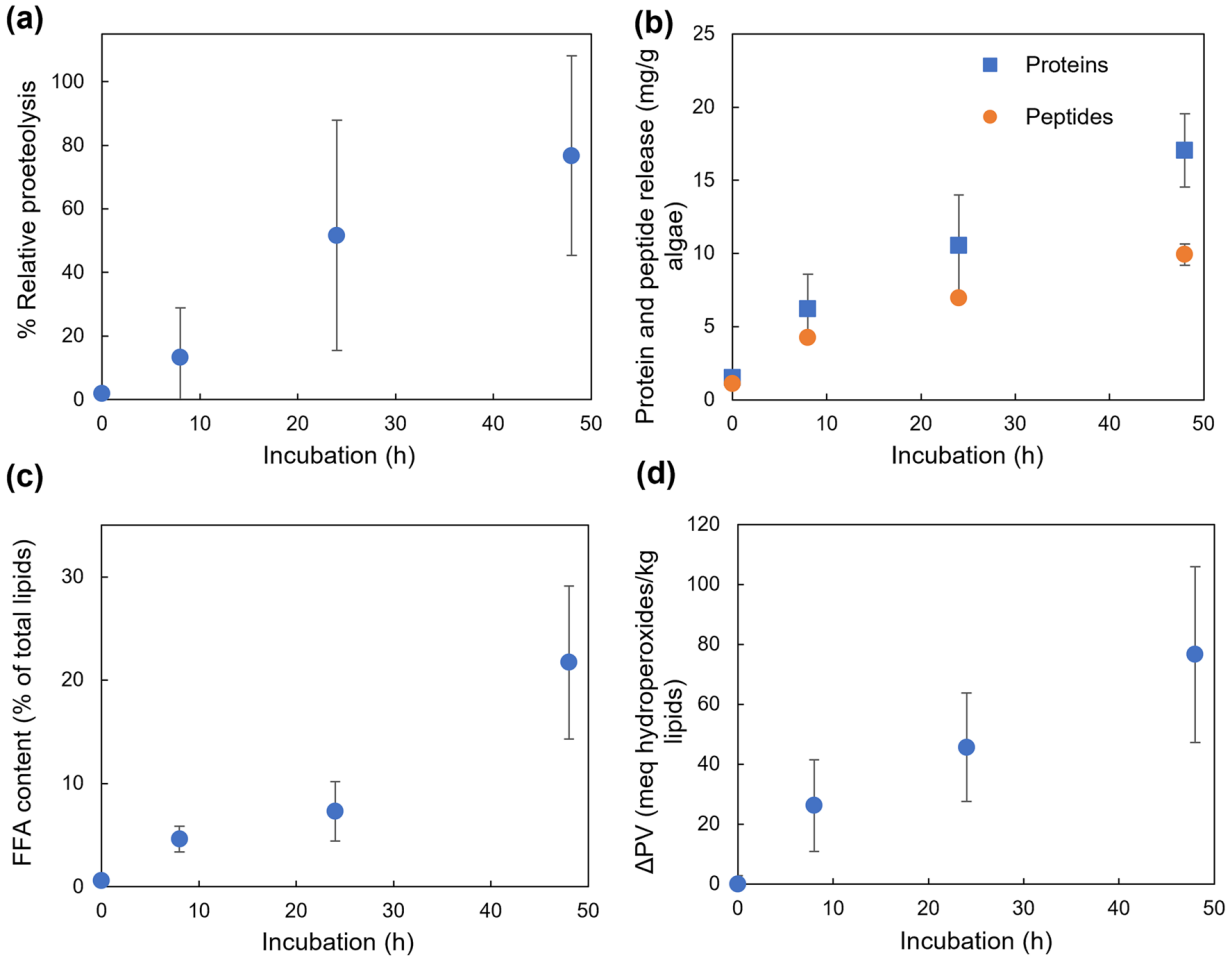
The effect of dark anoxic incubation on (**a**) the relative proteolysis of proteins as a % compared to day 0, **b** release of peptides and total protein (including peptides) from cells into the supernatant, and the (**c**) FFA content and (**d**) peroxide value (ΔPV) of the lipids. The data points and the error bars represent the average and standard deviation of duplicate measurements of triplicated experiments. Spearman correlation analysis returned (**a**) *ρ* = 0.822*, p* = 1E-3, (**b**) peptides: *ρ* = 0.982, *p* = 1E-5, and proteins: *ρ* = 0.928, *p* = 1E-5 (**c**) *ρ* = 0.928, *p* = 1E-5, (**d**) *ρ* = 0.928, *p* = 1E-5 confirming strong and statistically significant (*p* <  < 0.05) positive monotonic relationships with time for all aspects considered

As the first step, the effect of dark anoxic incubation on the total protein and lipid content was measured and the previous observations by Halim, et al. [8] were confirmed (Figure S1). The biochemical integrity of the protein and lipid content of the algal biomass has important practical implications. The effect of dark anoxic incubation on protein and lipid integrity was investigated by measuring the RP of the proteins (Fig. 1a), the release of proteins and peptides from the cells into the supernatant (Fig. 1b), and the free fatty acid content (Fig. 1c) and PV (Fig. 1d) of the lipids as a function of incubation time.

For the proteins, the RP increased with the increasing incubation time (Fig. 1a). Proteins and peptides progressively leaked from the cells into the supernatant (Fig. 1b), where the peptide content was measured after separating using a membrane cut off filter following which both the total proteins in the supernatant and peptides in the separated fraction were quantified using a modified Lowry assay capable of detecting both proteins and peptides [34] as described above in sect. Protein and peptide release during incubation. Initially, peptides were the majority of released proteinaceous material, reflecting their smaller size. As the incubation time increased, the peptide-to-protein ratio decreased suggesting the cells progressively became sufficiently permeable to release whole proteins/larger peptides.

As seen in Fig. 1c, the FFA content increased with increasing incubation time. As the total lipid content did not change during incubation (Figure S1), the increase in FFA suggests lipolysis is taking place during dark anoxic incubation. Notably, the FFA content is seen to increase dramatically between 24 and 48 h of dark anoxic incubation. The PV is an indicator of lipid peroxidation, which is detrimental to lipid quality. The PV increased approximately linearly with respect to incubation time (Fig. 1d). The increase in FFA content and PV demonstrates that dark anoxic incubation results in degradation of lipid quality, particularly beyond 24 h.

**Fig. 2:**
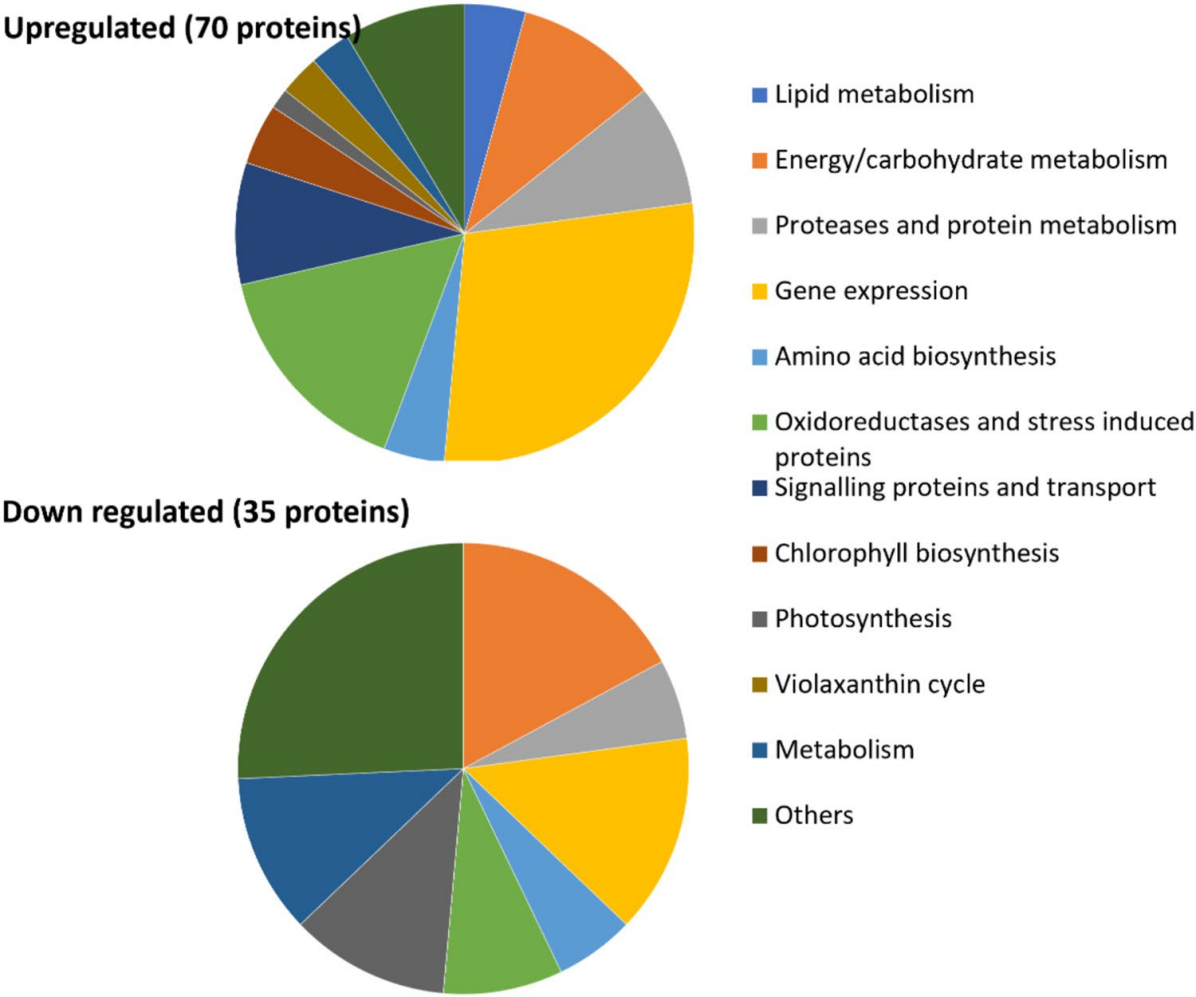
Functional groupings of proteins differentially expressed during dark anoxic incubation

Through the proteomic analysis, around 1700 proteins in the *Nannochloropsis* proteome could be identified. However, among the 1700 proteins, only around 170 were found to be differentially expressed (*p* < 0.05). Proteins involved in protein, lipid and carbohydrate metabolism were seen among these, in addition to the proteins associated with stress responses. Table S1 includes the proteins that were seen to be differentially expressed during the process of incubation, categorised based on their major cellular functions. Figure 2 presents the number of up- and downregulated proteins grouped by function.

As seen in Fig. 2, there were significantly changed proteins involved in a broad range of cellular functions including lipid metabolism, energy/carbohydrate metabolism, protein catabolism, amino acid biosynthesis, photosynthesis, chlorophyll biosynthesis, stress responses, protein translation and transcription, and signalling and transport. More of the proteases and proteins related to protein metabolism (transcription, translation and protein folding) increased than decreasing in abundance along with the enzymes related with oxidoreductase activity. The activity of the violaxanthin cycle seemed to be increased as two related enzymes were significantly upregulated. Oxidoreductase and stress response was another group affected during dark anoxic incubation. Considering Table S1, it is clear that there were many individual proteins within the functional groups that were variously up and down regulated. The implications of specific proteins of apparent importance will be discussed in the next section.

**Fig. 3:**
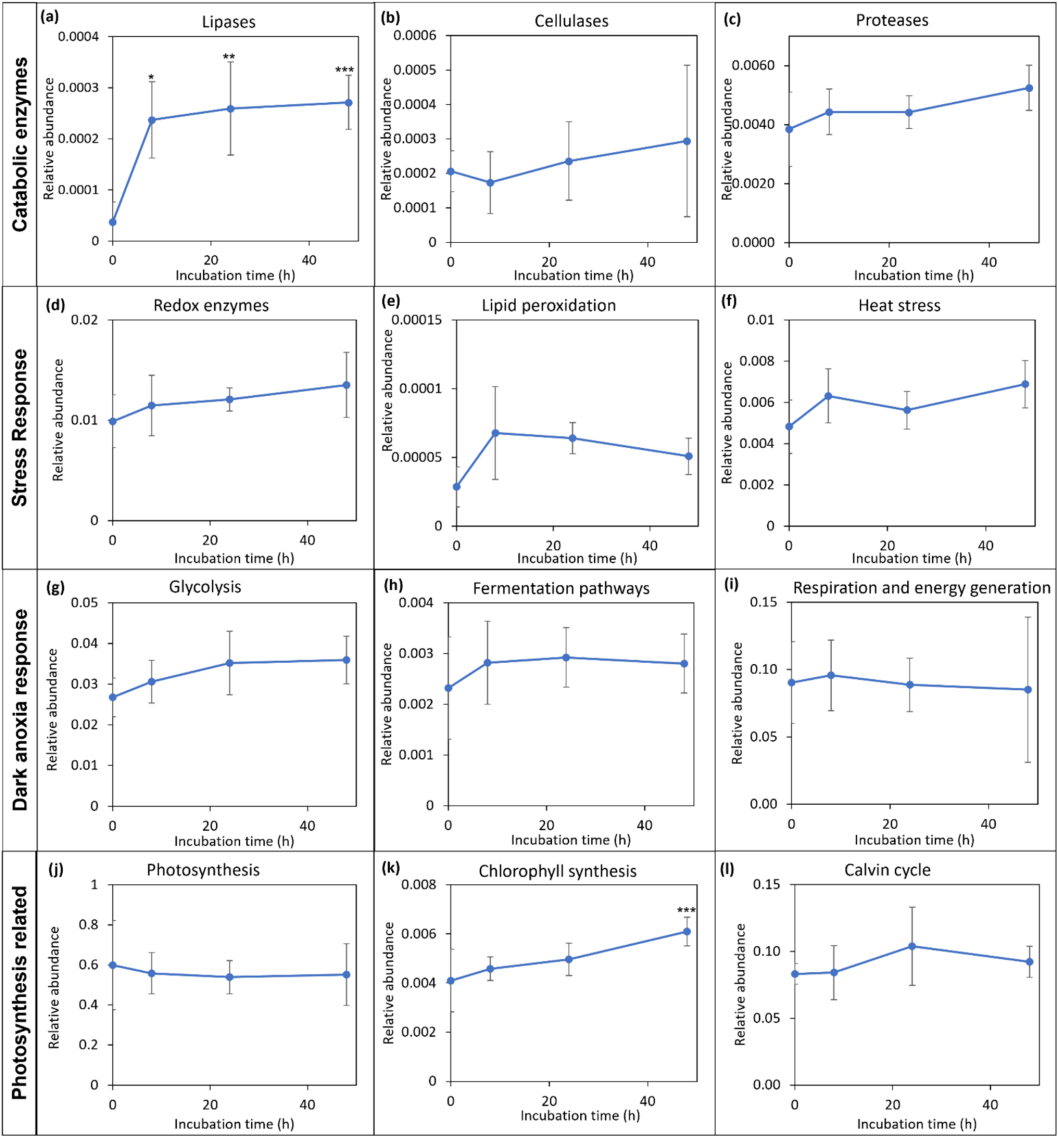
The effect of dark anoxic incubation time on the relative abundance of protein groups involved in the selected processes of interest. **a** Lipases, **b** cellulases, **c** proteases (sum of all proteases, peptidases and proteasome complex units), **d** redox enzymes (sum of all the enzymes/proteins having oxidoreducIe activity), (**e**) lipid peroxidation (sum of all lipoxygenases), **f** heat stress proteins (induced as a response to heat stress, with the function of these proteins mostly associated with protein folding and refolding), (**g**) glycolysis (proteins associated with the glycolytic pathway), **h** fermentation pathways (proteins identified to be involved in different fermentative pathways to maintain the redox balance of the cell), **i** respiration and energy generation (proteins involved in respiratory energy metabolism), **j** photosynthesis (proteins involved in photosynthesis including light harvesting complexes, photosystem I and II complexes and cytochrome proteins), **k** chlorophyll synthesis, **l** Calvin cycle (proteins involved in photosynthetic C fixation through the Calvin cycle). The proteins included in each of these groups and their associated data is included in supplementary information (Supplementary 2). Asterisk symbols represent statistically significant differences compared to the *t* = 0 time point

Apart from identifying the statistically significant proteins and protein groups that were differently abundant/expressed, the effect of incubation on the overall abundance of different protein groups of specific functions, irrespective of whether the individual proteins have changed or not, was considered important. From previous studies of *Chlamydomonas*, specific functional groups were seen to be affected by dark anoxic incubation [10, 12]. Hence the proteins showing relevance to different catabolic processes, energy production, photosynthesis and stress response, were manually selected and characterised. The sum of the relative abundance of individual proteins in each functional group was obtained and compared as a function of incubation time and is depicted in the following Fig. 3.

Statistically significant changes were observed only in the lipase and chlorophyll biosynthesis groups. However, looking at the overall trends, protein groups involved in catabolic and stress response activities such as lipase, proteases, cellulases, heat stress induced, and redox enzymes showed an overall increasing trend during incubation. There was a sudden 2–threefold increase in lipid peroxidation related enzymes after 8 h of incubation, which subsequently subsided. Of the protein groups involved in dark anoxia responses, glycolysis and fermentation pathways showed an overall increase, while the enzymes involved in respiratory energy generation decreased over the period of incubation. Photosynthesis related proteins progressively decreased, although interestingly the enzymes involved in chlorophyll synthesis showed an increasing trend. Generally, it can be observed that the cellular response within the first 8 h of incubation induction was more pronounced than later in the incubation period. This can likely be attributed to the significant loss of cell viability that occurred after 24 h of incubation (previously, more than 60% of the cells lost viability [8]). The loss of cell viability and associated cessation of metabolic activities coupled with uncontrolled protein degradation led to substantial variations in cellular protein signals between sample replicates at 48 h point. Some protein groups (such as cellulase, respiration and energy generation) displayed particularly high levels of variations in their relative abundance. The significance of these protein groups and the variation of the relative abundance will be discussed in the following discussion.

## Discussion

### Effect of dark anoxic incubation on protein and lipid integrity

The observed increases in the peptide content and cellular FFA (Fig. 1a and c) could be a result of either or both of two processes. First, the degradation of lipids and proteins could be a result of an active cellular response to the stress conditions they are undergoing. In this case, the cells would actively express proteolytic and lipolytic enzymes, possibly as part of cellular reassembly or to maintain energy flux under the stress conditions. Alternatively, it could be an inadvertent effect of the release of compartmentalized or membrane-bound proteases and lipases in the cells as a result of global intracellular degradation. This would be consistent with the transmission electron microscopy images obtained in a previous study, which revealed that the cells’ organelles slowly lost their integrity over the incubation time period [8].

Proteasomes and proteases function in cells by degrading misfolded or unwanted proteins. The stress conditions provided (high temperature and anoxic conditions) could promote protein misfolding and aggregation, or result in the redundancy of certain enzymes (e.g. those involved in photosynthesis), therefore requiring the degradation of such proteins and overexpressing of proteolytic enzymes [14, 35]. The observed increase in two proteases, one peptidase and two ubiquitin enzymes (Table S1) could suggest that the cells are actively increasing the metabolism and degradation of unwanted proteins. In particular, the 26 s protease regulatory subunit 8 increased 13-fold in the first 8 h, after which it remained constant. Two ubiquitin enzymes also increased in abundance, suggesting the proteasome-ubiquitin pathway is activated. This 26 s proteasome pathway is located in the cytoplasm and nucleus and is involved in degrading misfolded and unwanted proteins [36]. However, degrading the proteins through the ubiquitin–proteasome pathway requires energy, which as discussed below is highly limited during dark anoxic incubation. Also, while the overall content of proteases available in the cells was seen to increase during prolonged incubation (Fig. 3), it was quite minor (*ca* + 10%).

Apart from this, the ATP-dependent zinc metalloprotease related to cell division and protein catabolic process also increased in abundance (Table S1). Most importantly, this was the most abundant protease observed in all the protein samples irrespective of the incubation time. Such zinc metalloproteases have previously been identified to have the ability to degrade the extracellular matrix proteins of the microalgae cell wall, eventually leading to cell autolysis in *Chlamydomonas reinhardtii* [37–39]. This protease could be playing a key role in the above suggested cellular degradation. Abiotic stresses such as light limitation in microalgae have induced proteases that led to autocatalyzed cell death [14, 40]. In a previous study, the unicellular chlorophyte *Dunaliella tertiolecta* activated apoptotic cell death when placed in prolonged darkness, which was seen to be related to an increase in protease activity and the induction of certain proteases [14, 40, 41]. These mainly consisted of cell death-associated proteases or caspases, whereas the inhibition of such proteases had prevented the appearance of reactive oxygen species and apoptosis under dark conditions [41]. Similarly, heat stress on eukaryotic microalgae *Chlorella saccharophila* has also led to an increase in apoptotic-like cell death, which was significantly reduced following the inhibition of caspase like enzymes [42].

Importantly, the progressive increase in the RP of the proteins during prolonged dark anoxic incubation (Fig. 1a) indicates an imbalance between protein degradation and assembly. In healthy cells, protein degradation is balanced by reassembly of correctly folded proteins needed by the cells. The high degree of unbalanced proteolysis suggests an uncontrolled process. It is possible that the initial spike in 26 s protease regulatory subunit 8 and the zinc metalloprotease could have accelerated this to some extent. Overall, the evidence supports the idea that protein degradation was initially promoted by proteases expressed as part of apoptotic cellular degradation resulting from the dark, anoxic and heat shock provided to the cells, and then accelerated by unregulated proteolytic activity.

As seen from Fig. 1b, the protein content present in the supernatant prior to incubation was mostly peptides. As the incubation time increased, more proteins were released into the supernatant, which were initially also peptides. This reflects the higher permeation rate of peptides relative to whole proteins. Despite the continued progression of proteolysis (Fig. 1a), whole proteins eventually leaked out from the cells at a higher rate than peptides, presumably due to the continued loss of cell membrane integrity. Compromised membrane permeability was another effect of caspase like proteases leading to apoptotic cell death [43].

Similar to the protein hydrolysis, the observed lipolysis (as evidenced by the constant total lipid content and increasing FFA) could be an inadvertent consequence of cell degradation or a result of deliberate cellular action. In contrast to the small increase in protease concentration, the total amount of lipases in the cells increased considerably (*ca* + 600%) during dark anoxic incubation (Fig. 3). In particular, the PLA2c domain-containing protein that has phospholipase activity was found to increase in abundance by over 40-fold during dark anoxic incubation (Table S1). Previously, similar dark storage of intact *Nannochloropsis* sp. slurries resulted in an increase in the FFA content, with a concurrent decrease in the polar lipids with two fatty acids suggesting the lipolysis of membrane lipids [21]. As phospholipases act on the membrane phospholipids, the increase in the free fatty acid content (Fig. 1b) is consistent with lipolysis of membrane lipids and a loss of membrane integrity. The activity of a phospholipase has been observed to initiate chemical defence in the diatom *Thalassiosira rotula* after cell damage [44]. This observed increased activity of the phospholipase could be part of a similar defence strategy by the *Nannochloropsis* sp. cells.

The loss of membrane integrity resulting from phospholipase activity is consistent with the increased release of whole protein during incubation. Lipolysis, or the formation of FFA, was previously attributed to be a sign of microalgae (*Nannochloropsis* and *Isochrysis*) cell damage or presumably cell death-induced mechanisms caused by different stress conditions [21, 22]. Such stress conditions induce lipase activity or make cells fragile and accessible to endogenous lipases, whereas rapid heat treatments designed to inactivate these endogenous lipases have increased lipid stability [45, 46]. Several previous studies have observed changes in the total fatty acid content under dark anoxia [10], under dark stress [47], and under combined dark and cold stress conditions [48]. However, dark anoxic incubation of *Nannochloropsis* did not seem to significantly affect the total lipid and fatty acid content, lipid class distribution or overall FAME composition [8]. This is consistent with other studies on microalgae such as *Nannochloropsis salina* [9], *Tetraselmis suecica* [49] *D. tertiolecta* where the fatty acid content has not been significantly affected during dark anoxic conditions.

Polar lipids in microalgae have been found to be more sensitive to lipolysis than TAGs [45, 50]. This could be partly due to the relative inaccessibility of the TAG molecules (largely protected within lipid bodies). In *Nannochloropsis* sp. TAGs have been found to consist mostly of saturated fatty acids whereas polar lipids are richer in unsaturated fatty acids including PUFAs [51, 52]. Membrane lipids, especially the unsaturated fatty acids, undergo changes under biotic and abiotic stresses, during the induction of defence reactions. Changes in the degree of fatty acid unsaturation have also been observed under abiotic stress conditions [53].

The main cellular functions of lipolysis or lipid degradation include production of carbon and energy for growth, remodelling membrane lipid composition, and generation of signalling molecules [54]. Lipids can be used to generate energy in dark conditions via lipolysis followed by fatty acid beta oxidation in the presence of oxygen [54]. However, the lack of oxygen in the environment prevents fatty acid beta oxidation. The stasis of the total FA content in *Nannochloropsis* during dark anoxic incubation [8] suggests the lipids (both TAGs and membrane lipids) are not hydrolysed for energy generation (in which case the overall content would be decreased [15]), but rather an inadvertent effect of cellular degradation, and possibly as a defence mechanism.

Membrane lipids have been shown to be sensitive to cellular heat stress. Under heat stress, the fluidity of the membrane increases, which can be actively countered by the cells via increasing the degree of fatty acid saturation to maximise hydrophobic interactions [55]. Therefore, the observed increase in the FFAs can be associated with the spike in the phospholipase enzymes that act on the membrane lipids as a stress response trying to remodel the membrane lipids.

Lipid peroxidation is important for lipid quality and is linked with the amount of reactive oxygen species (ROS; superoxide radicals (O_2_^−^), hydroxyl radicals (^−^OH) and hydrogen peroxide (H_2_O_2_)) present and the presence of FFAs and lipoxygenase enzymes which can accelerate lipid peroxidation. As seen from Fig. 3, although the increase was not statistically significant, there was an apparent increase in the lipoxygenases observed during dark anoxia that could potentially promote lipid peroxidation (Fig. 1d). Therefore, the activity of the phospholipase is likely to play a major role in both FFA formation and lipid peroxidation.

As described earlier, the induction of several stress conditions resulted in degradative effects on the proteins and lipids. Therefore, the following section involves a detailed discussion on the observed differences in the cell proteome and the potential responses of the cells including the response to dark anoxia and heat stress, that could provide insights into the mechanism used to survive these stress conditions and trigger the observed biochemical degradation.

### Cellular stresses during dark anoxic incubation

Dark anoxic incubation subjects the algae cells to three main stresses: (i) dark conditions that prevent photosynthetic carbon assimilation and energy generation, (ii) anoxic conditions that prevent oxidative respiration, and (iii) heat stress.

When microalgal cells are subjected to high temperature stresses, an accelerated expression of heat responsive genes and heat shock proteins has been observed [56, 57]. As an example, the expression of heat shock proteins were seen in the red alga *Cyanidioschyzon merolae* and the green alga *Chlamydomonas reinhardtii* when subjected to high temperatures (36 °C for *Chlamydomonas reinhardtii* and 55 °C for *Cyanidioschyzon merolae*) relative to their optimal growth temperatures (22 °C and 42 °C, respectively) [57, 58]. Although the threshold temperature for heat stress in the studied strain of *Nannochloropsis* has not been determined, *Nannochloropsis* naturally grows in similar climate conditions as does *Chlamydomonas*, which could suggest that activation of heat response could occur at the incubation temperature of 37 °C. The exact role of elevated temperature during the current dark anoxic incubation is not clear, however there are some signs of heat stress in the proteome. In particular, a heat shock protein (A0A4D9D7K1) involved in protein folding (Table S1) was overexpressed indicating an active metabolic adjustment to counter these effects.

Two proteins in the violaxanthin cycle, violaxanthin de-epoxidase related protein (A0A4D9DAF3) and zeaxanthin epoxidase protein (A0A4D9CSI8) were upregulated under dark anoxic conditions (Table S1). This response aligns with observations in other photosynthetic organisms, where the violaxanthin cycle can be activated by various abiotic stresses, even in darkness [59]. This mechanism may prepare *Nannochloropsis* for rapid photoprotection upon re-illumination or re-oxygenation, similar to the enhanced xanthophyll cycle induction seen in C. reinhardtii following dark anaerobiosis [60]. This upregulation may also contribute to membrane stabilization and antioxidant defense, crucial for maintaining cellular integrity under anoxic stress and during subsequent environmental transitions.

Beyond the direct effects of heat and other abiotic stresses, cells need energy if they are to actively metabolise and express new proteins and recycle the misfolded or damaged proteins. Therefore, the following section discusses the energy production mechanism during dark anoxic incubation, and their link to the other two stresses – darkness and anoxia.

### Energy production in Nannochloropsis sp. during dark anoxia

To maintain cell integrity and functioning, a means of cellular energy generation is required. Under normal circumstances, microalgal cells produce energy in the form of ATP via photosynthesis when light is available, and by respiration of carbon reserves (e.g., TAG or starch) using O_2_ as the main electron acceptor during periods of darkness. However, under dark anoxic conditions, the algae cells are not able to undertake either of these energy generating processes due to the simultaneous lack of both light and oxygen. In the absence of oxygen or other terminal electron acceptors, many microorganisms can resort to substrate level phosphorylation using fermentation pathways, which catabolise organic compounds with no net oxidation.

Fermentation metabolism has been confirmed and studied in detail in the model microalga, *Chlamydomonas reinhardtii* [61]. Under dark anoxic conditions *Chlamydomonas* can maintain cellular energy generation and redox balance by fermenting stored starch via glycolysis into end-products including formate, acetate, ethanol, CO_2_ and H_2_ [61]. Fermentation metabolism has yet to be confirmed in *Nannochloropsis*, but was previously proposed in our previous studies of dark anoxic incubation of this alga [8]. One complication however, is that for glycolysis to take place, a carbohydrate substrate should be available to ferment. Unlike *Chlamydomonas* that uses starch as its cellular energy and carbon reserve, *Nannochloropsis* is primarily a lipid (TAG) accumulator. As TAG has a much higher degree of reductance than starch, it cannot be readily fermented. However, in addition to TAG *Nannochloropsis* can accumulate glucans (chrysolaminarin) as storage carbohydrates and their cell walls contain cellulose [4, 62]. It was previously observed that the cellulose layer in *Nannochloropsis* sp. was consumed during dark anoxic incubation, and it was proposed that the cellulose may have been metabolised as part of fermentation metabolism [8]. For this to occur, there needs to be cellulases present in the cells that can hydrolyse the cellulose and glucans present in the *Nannochloropsis* cells. In the present study we in fact observed a few glycosidic hydrolases including betaglucosidase, endoglucanase, and lactase in the *Nannochloropsis* proteome (Supplementary 2). The overall abundance of proteins with cellulase activity was seen to increase during incubation (albeit, not statistically significantly, Fig. 3), with alpha glucosidase and endo glucanase found to be significantly upregulated (Table S1). These observations coupled with the thinning of the cellulose layer of the cell walls observed by Halim, et al. [8], provides further evidence that *Nannochloropsis* cells degrade the cellulose component of their walls as a resource for subsequent glycolysis.

**Fig. 4:**
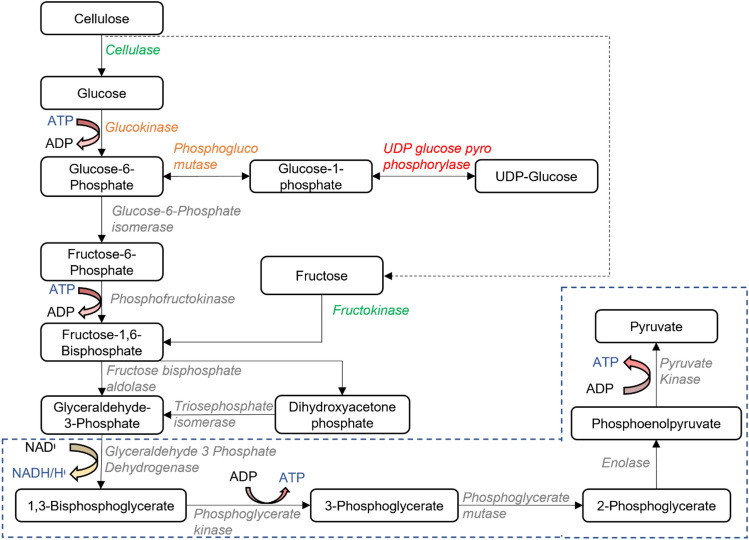
Localisation of the main enzymes and intermediates identified in this study involved in ATP production through the glycolytic pathway. Green represents significantly upregulated enzymes, grey upregulated but not significantly, orange down-regulated but not significantly, and red significantly down regulated enzymes. Blue dashed line indicates the reactions in the lower glycolytic pathway and the rest belongs to the upper glycolytic pathway

Following cellulose hydrolysis, the glycolytic pathway includes several steps catalysed by different enzymes that lead to the generation of the central metabolite pyruvate. Figure 4 summarises the different steps involved in glycolysis along with the associated enzymes found through the proteomic analysis in this study. This comprises of two main stages: upper glycolysis which is energy consuming, and lower glycolysis which is energy yielding. The upper glycolytic pathway produces two glycerol-3-phosphate (G3P) molecules, consuming one glucose molecule and 2 ATP molecules that will be eventually converted to two pyruvate molecules along with 4 ATP and 2 NADH via the lower glycolytic pathway. Therefore, the glycolytic pathway is able to produce 2 ATP that can be used as energy reserves for cellular processes [11]. The key steps in the glycolytic pathway that generate ATP are catalysed by the enzymes phosphoglycerate kinase (PGK) and pyruvate kinase (PK) resulting in the production of two pyruvate molecules.

Importantly, all of the different enzymes involved in glycolysis were observed in the *Nannochloropsis* proteome. Apart from the cellulases mentioned earlier, only fructokinase (FK) catalysing the conversion of fructose to fructose-1-phosphate (F1P) was significantly upregulated during dark anoxic incubation (Table S1). However, as fructose was not previously found to be abundant in *Nannochloropsis sp.* [4, 8], the reason for this is unclear.

Generally, the limiting steps in the glycolytic pathway have found to be those catalysed by glucokinase (GK) and phosphofructokinase (PFK), as they consume energy for the forward reaction [63]. In the present study, GK was downregulated while PFK was upregulated, although both changes were not statistically significant. Overexpression of PFK has previously led to an increased glycolytic activity in the diatom *Thalassiosira pseudonana* [63], and it could be that GK is not the rate-limiting step in *Nannochloropsis* sp. glycolytic pathway. Further in-depth studies are required to determine the rate-limiting steps in the *Nannochloropsis* glycolytic pathway.

Considering the other enzymes involved in the glycolytic pathway, although the difference was not statistically significant, most of the enzymes showed an increase in their fold change when compared with the unincubated, fresh cells. This suggests *Nannochloropsis* sp. cells attempt to upregulate glycolytic metabolism to produce the energy required for cellular processes in response to the stress conditions. Reciprocally, Uridine diphosphate (UDP) glucose pyrophosophorylase, which is needed for glucose activation in the synthesis of cellulose and other glucose polymers [4], was downregulated. The absence of photosynthetically fixed carbon during dark anoxia reduces the need for this enzyme for cellulose or chrysolaminarin synthesis.

Among the statistically unchanged enzymes, fructose bisphosphate aldolase (FBA), glyceraldehyde 3 phosphate dehydrogenase (G3PDH), pyruvate hydratase (PHy) and pyruvate kinase (PK) nonetheless showed an increasing trend during incubation (Figure S2). FBA has been found to be rate-limiting in upper glycolysis in *E.coli* [64]. The conversion of NAD + to NADH in the lower glycolytic pathway catalysed by the enzyme G3P leads to an imbalance in the redox state of the cell. Therefore, the pyruvate produced must be metabolised to regenerate the oxidised NADH or FADH2 and to produce more ATP in subsequent processes [11]. The following section discusses on the different fermentative and other pathways that were identified in the *Nannochloropsis* proteome that can be used to maintain cellular redox balance.

### Fermentation and other related pathways for maintaining cellular redox balance

**Fig. 5:**
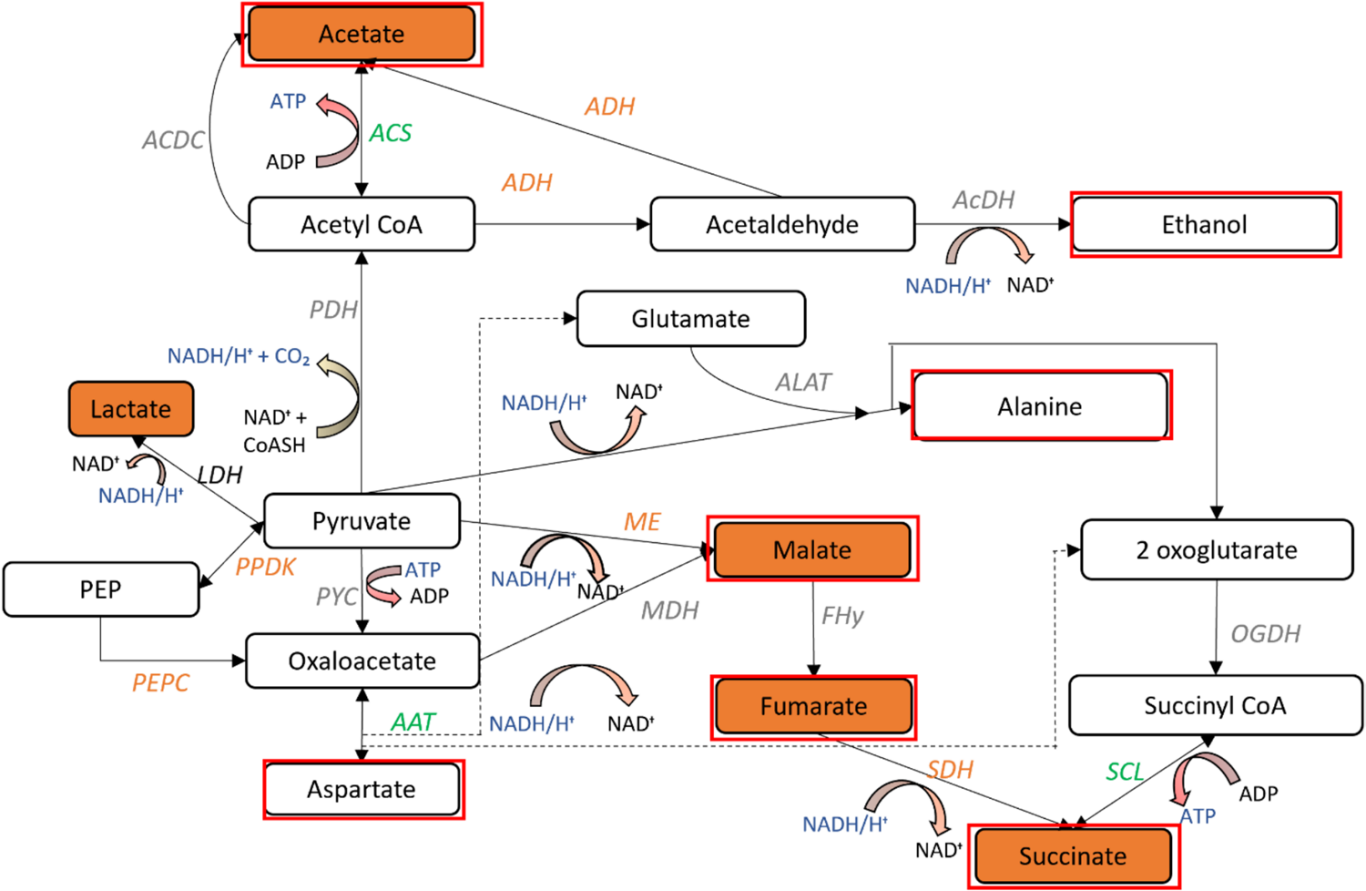
Identified enzymes and associated fermentative pathways in *Nannochloropsis* sp. under dark anoxia. Fermentative end products associated with the identified pathways are indicated by red boxes, with filled orange boxes representing end products that were detected during dark anoxic incubation of *Nannochloropsis* sp. in our previous study [8]. PPDK Pyruvate phosphate dikinase, PDH Pyruvate dehydrogenase, PYC Pyruvate carboxylase, ACS Acetate synthase, ACDC Acetyl CoA deacylase, ADH Aldehyde dehydrogenase, AcDH Alcohol Dehydrogenase, ALAT Alanine aminotransferase, CS Citrate synthase, ME malic enzyme, OGDH oxoglutarate dehydrogenase, SCL Succinyl CoA lyase, SDH Succinate dehydrogenase, FHy fumarate hydratase, MDH Malate dehydrogenase, AAT Aspartate aminotransferase, LDH Lactate dehydrogenase. Green: significantly increased. Grey: increased but not significantly. Orange: decreased but not significantly Black: not found

As discussed in the previous section, under dark anoxic conditions *Nannochloropsis* cells are proposed to undertake glycolysis to produce energy required for cellular metabolism. In the absence of oxygen needed to reoxidise the NADH to produce NAD + via the citric acid /Krebs cycle fermentative pathways are required which lead to the formation of various organic acids, alcohols and gases such as H_2_ and CO_2_ depending on the pathways undertaken by the cells [8, 12]. In eukaryotes like *Nannochloropsis*, fermentation involves the conversion of the pyruvate produced during glycolysis into a range of end-products including ethanol, lactate, acetate, succinate and propionate, via intermediates such as acetyl CoA [11]. Figure 5 summarises all the fermentation and associated pathways *Nannochloropsis* cells are proposed to undertake during dark anoxic incubation based on proteomic data obtained. Each of these pathways is discussed in detail in this section.

The conversion of pyruvate into acetyl CoA is a crucial step in carbon energy metabolism, and this can be catalysed by the pyruvate dehydrogenase complex (PDH), pyruvate:ferredoxin oxidoreductase (PFO) [11], or pyruvate formate lyase (PFL) [12], of which only the pathway through PDH was observed in *Nannochloropsis* based on our observations through proteomic analysis. Neither PFO nor PFL could be found in the proteome of *Nannochloropsis*. The PDH enzyme complex did not show a significant increase during incubation, but the average relative abundance of the proteins did increase slightly by around 26 ± 5% (Figure S3).

Acetyl CoA can be further metabolised into ethanol through alcohol dehydrogenase (AcDH) and aldehyde dehydrogenase (ADH) (also called ethanolic fermentation), as has been confirmed in *Chlamydomonas* [12]. During dark anoxic incubation of *Nannochloropsis*, AcDH (Figure S3) was seen to be upregulated, although the difference was not statistically significant. Aldehyde dehydrogenase was present, but not changed significantly. The presence of these enzymes indicates the ability of *Nannochloropsis* sp. cells to use these pathways. The lack of upregulation of these enzymes suggests that this pathway was not favoured. This is consistent with our previous study on dark anoxic incubation of *Nannochloropsis* in which ethanol was not detected as a fermentation end product [8]. There are reports showing the accumulation of esters following long storage of *Nannochloropsis* and *Isochrysis* under dark conditions [21], which could indicate that any ethanol that was produced during dark anoxic metabolism was consumed in reactions with lipids to produce esters. Apart from the ethanol fermentation pathway, acetyl CoA can be converted to acetate through five different pathways in eukaryotes to convert ADP to ATP. These include acyl CoA thioesterase (ASCT) pathway, acetate succinate CoA transferase (ACST) pathway [11] Acyl CoA deacylase pathway, acyl CoA synthetase (ACS) pathway and the phosphotransacetylase (PTA) and acetate kinase (ACK) pathway [12]. Among these the PTA, ACK and ASCT enzymes were not found in the *Nannochloropsis* proteome. The PTA ACK pathway was the dominant pathway under dark anoxic conditions in *Chlamydomonas reinhardtii* [65]. However, during incubation of *Nannochloropsis*, only the ACS pathway and the ACDC pathway were observed, with ACS significantly overexpressed (Fig. 5, Table S1), similar to previous observations in *Chlamydomonas* cells [12].

The next common pathway of fermentation is the lactate fermentation, catalyzed by lactate dehydrogenase (LDH) [66]. Lactic acid was detected in our previous study in which *Nannochloropsis* was subjected to dark anoxic incubation [8] indicating that lactate as well as ethanolic fermentation were used to reoxidise NADH. LDH is indeed present in the *Nannochloropsis* proteome found through a protein search in the UniProt database [27], however it was not detected in the proteomic samples in the present study. It is possible that the protein was expressed at low levels and was present below the limits of detection.

A rapid increase in alanine aminotransferase (ALAT) [67] and a resultant accumulation of alanine [66] has been observed in plant cells under anoxia. ALAT catalyses the transamination of pyruvate to alanine and conversion of pyruvate and glutamine to alanine and 2-oxoglutaric acid, playing a key role in carbon and nitrogen metabolism in plants [66]. It has been proposed that functions of ALAT during anoxic conditions are to reduce cellular acidity [65], prevent the loss of carbon through the fermentation pathways, and to yield ATP through substrate level phosphorylation [68]. In addition, the produced 2-oxoglutarate is converted to succinyl CoA catalysed by oxoglutarate dehydrogenase (OGDH) and succinyl CoA to succinate via succinyl CoA lyase (SCL) releasing another ATP molecule [67]. In this study, all of these enzymes were found to be present, with ALAT and SCL found to increase in abundance during dark anoxic incubation (the increase in SCL but not ALAT was statistically significant). Although the presence of alanine was not previously verified [8], the presence of all necessary enzymes suggests this pathway could be active in *Nannochloropsis*.

The two pathways discussed above, a part of the reverse TCA cycle and part of the TCA cycle starting from 2 oxoglutarate, both lead to the production of succinate, which was previously observed in dark anoxic incubation of *Nannochloropsis* [8]. It was proposed previously that succinate biosynthesis occurs through the reductive TCA cycle in *Euglena gracilis* under dark anoxic conditions [69]. For this, aspartate is converted to oxaloacetate (OAA) by aspartate aminotransferase (AAT), which is then reduced to succinate. A rapid drop in aspartate coupled with an increase in alanine and succinate has previously been observed during dark anaerobic treatment of the microalga *Selenastrum minutum* [70]. The presence of all required enzymes including a significant increase in AAT (Table S1), suggests that this is another possible pathway used by *Nannochloropsis* during dark anoxia.

Under anaerobic conditions, a partial reductive TCA cycle where OAA is reduced to malate, fumarate and succinate was shown previously for *Euglena gracilis* under dark anaerobic conditions [71]. OAA is reduced to malate by cytosolic malate dehydrogenase (MDH) to reoxidise NADH [11]. The malate can subsequently be metabolized to succinate through fumarate via part of the reverse TCA cycle [69]. Fumarate reduction to succinate results in the oxidation of NADH and ATP production, hence is a productive anaerobic metabolism. This pathway seems to be active during dark anoxic incubation in *Nannochloropsis*, with a slight increase in MDH found during incubation, along with the presence of fumarate hydratase and succinate dehydrogenase (Figure S3), as well as detected malate, fumarate and succinate in our previous study [8].

In summary, it can be concluded that during dark anoxia *Nannochloropsis* cells produce ATP through glycolysis and a range of fermentative pathways. However, it should be noted that the amount of energy produced during dark anoxia is significantly lower than that which can be produced by photosynthesis or by aerobic respiration of cellular carbon reserves in the presence of oxygen. This represents a secondary stress on the cells, which need to limit energy usage to support only the most critical functions.

### Effect of dark anoxic incubation on photosynthetic and light harvesting proteins

Previously, when *Nannochloropsis* sp. cells grown in high light were transferred to low light conditions, the volume of the chloroplasts of the cells, the photosynthetic unit (PSU) density, thylakoid surface area and the pigmentation all increased representing a 1.7-fold increase in the overall light harvesting potential [72]. This demonstrates that, when cells still have a photosynthetic energy supply, they can compensate for limited light availability by diverting resources towards increasing their light harvesting ability.

In contrast, due to the complete lack of light during incubation, the cells cannot perform photosynthesis, and as discussed above, have a limited energy supply. While cells could potentially reduce resources devoted to unused photosynthetic apparatus, photosynthetic microalgae are naturally subjected to cyclical light availability and should not be programmed to completely deconstruct its photosynthetic apparatus during periods of darkness. In line with this, *Chlamydomonas* has been found to retain but downregulate its photosynthetic apparatus and enzymes related to chlorophyll synthesis during dark anoxia [10]. In comparison, in this study, while there was a reduction in two light harvesting proteins (W7TX20, W7T8I0), there was an increase in magnesium chelatase and geranylgeranyl reductase, two major enzymes involved in the chlorophyll biosynthetic pathways (Table S1).

Photosynthetic pigments are a critical component of photosynthetic apparatus. Halim, et al. [8] observed the chlorophyll a content in *Nannochloropsis* sp. to be degraded to pheophytin a (which is chlorophyll a lacking a Mg^+2^ ion) during dark anoxic incubation. As discussed above, the elevated temperature and dark anoxic environment stress the cells and limit energy availability. Chlorophyll degradation could be due to uncontrolled reactions with organic acids produced intercellularly due to fermentation or as an action of chlorophyllide oxygenase present in the cells [8, 73]. It has been observed that acidic conditions and high temperatures result in the degradation of chlorophyll a to pheophytin a [74, 75]. To compensate for this degradation, cells can try to produce more chlorophyll.

Apart from these, other major photosynthetic related enzymes including the light harvesting complexes and Photosystem I and II related proteins showed a slightly decreasing trend although the difference was not found to be significant (Fig. 3) similar to that previously observed in *Euglena gracilis* [76]. Light harvesting proteins were previously seen to be down regulated under dark conditions [77], similarly two light harvesting proteins (W7TX20, W7T8I0) showed a significant decrease in abundance (Table S1). There was no significant change in ATP synthases, consistent with what has been previously been observed in other microalgae (*Fragilariopsis cylindrus* and *Chlamydomonas reinhardtii*) [10, 77]. The PSI reaction subunit increased in abundance (Table S1), although unexpected similar observations have previously been observed [68, 78]. None of the proteins related to the Calvin Benson cycle, used for carbon fixation and light-dependant reactions, were significantly changed over the dark anoxic incubation period. Thus, based on these observations, it appears that as a photosynthetic microorganism, *Nannochloropsis* sp. tries to maintain their photosynthetic machinery even during prolonged dark anoxic stress conditions.

### Effect of dark anoxic incubation on other major cellular metabolic processes

Apart from the above discussed energy generation and stress responses in the cells, the enzymes related to protein synthesis, including transcription, translation and protein folding were significantly increased (Table S1). This is consistent with a previous study on *Chlamydomonas* in which transcripts encoding for transcription/translation regulation factors were seen to be increased during dark anoxia [12]. The increased levels of protein synthesis enzymes, despite the limited energy availability, may be a result of retooling in response to dark anoxic stresses. These stresses appear to trigger multifaceted cellular adaptations that require retooling of metabolic pathways, leading to an increased requirement for the synthesis of new/different proteins.

Signalling and transport proteins are required for cellular metabolism and were also affected during dark anoxic incubation (Table S1). The up-regulation of signalling proteins in response to different stress conditions has previously been observed in plants as well as microalgae such as *Chlamydomonas* [10, 79], where they play a major role when acclimating to stress conditions. The accumulation of amino acid transporters (A0A4D9CTY7) and several transmembrane transporters (A0A4D9CQ73, A0A4D9CY02, A0A4D9D9I1) (Table S1) may be related to cell response to the stress conditions. For example, fermentation end-products may need to be transported and excreted out from the cells in order to prevent acidosis [80]. UDP sulfoquinovose synthase enzyme, which is responsible for the formation of the membrane lipid sulfoquinovosl diacylglycerol (SQDG), was upregulated during dark anoxia (Table S1), similar to what was observed in diatoms [77]. SQDG is important for maintaining thylakoid membrane integrity and protecting the functionality of ATP synthase [77].

### Mechanistic summary

**Fig. 6:**
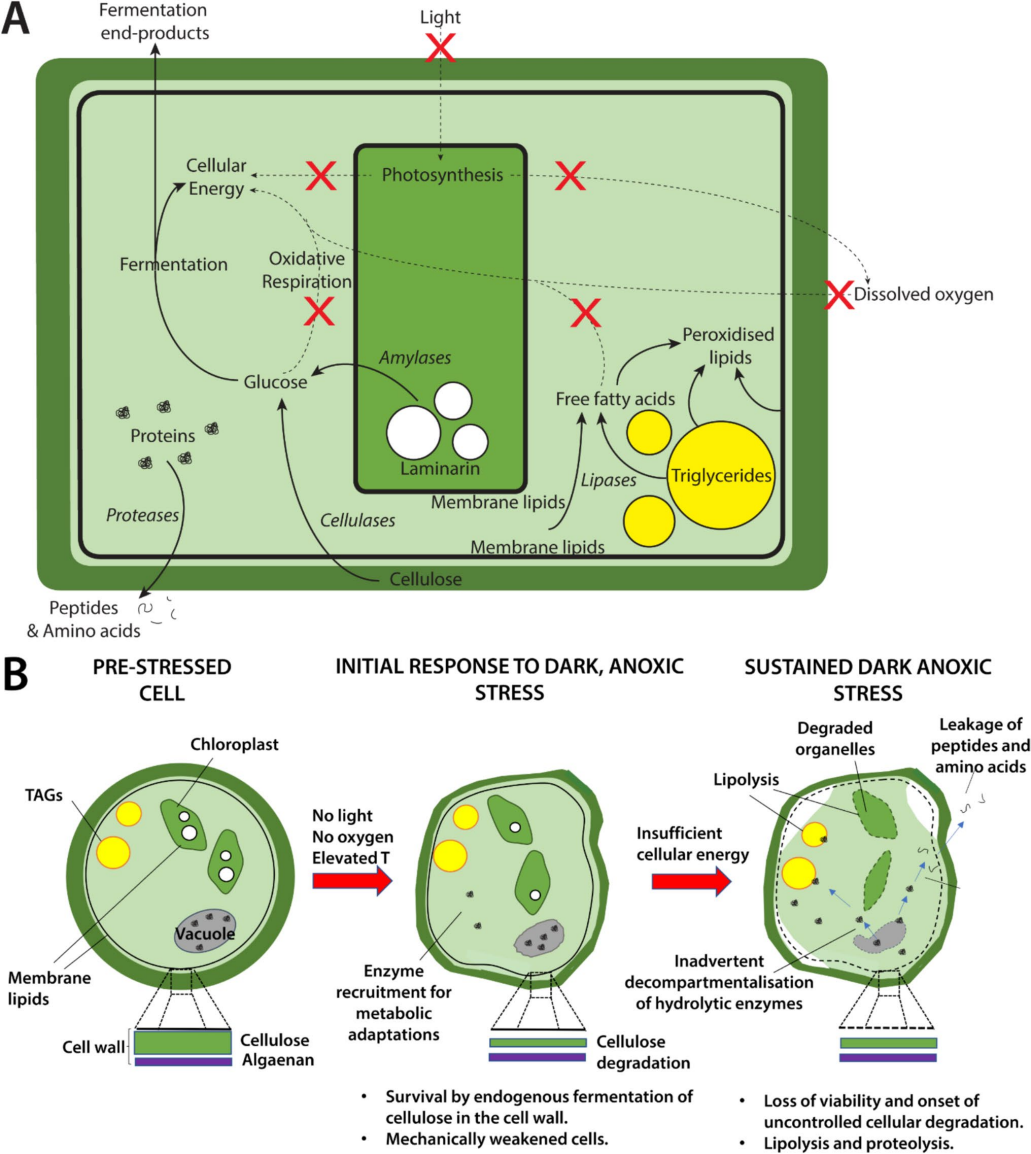
Graphical summary of the proposed mechanistic responses of *Nannochloropsis* to dark anoxic incubation at 37 ℃. **A** The major catabolic processes occurring in cells that are deprived of light and oxygen. Red crosses and dashed arrows represent catabolic pathways that are not available under dark anoxic conditions. **B** Progressive physiological and biochemical effects of dark anoxic stress

Figure 6 illustrates the major proposed mechanistic responses of *Nannochloropsis* to dark-anoxic incubation. These mechanisms have been assembled by combining findings from the current study with those from our previous studies [8, 15]. Figure 6A highlights the major catabolic processes that occur when the cells are deprived of light and oxygen. The lack of light prevents photosynthetic energy generation, forcing the cells to generate cellular energy by endogenous heterotrophy. However, as oxygen is not available to drive oxidative respiration of lipids or carbohydrates, the cells must use fermentative pathways involving depletion of cellular laminarin (a form of starch) and cellulose reserves (thereby thinning and weakening the cell wall) and the release of fermentation end-products such as ethanol and organic acids. Subsequently, intracellular hydrolytic enzymes are released during cellular breakdown that result in the formation of peptides and amino acids that can leak from the cells, and free fatty acids.

Figure 6B shows the progressive effects of the stresses of dark anoxia on the physiology and biochemistry of the cells. Within the first 24 h of dark anoxic incubation, cellular energy demands could be met by breaking down cellulose and laminarin. This depleted the cell walls (by almost 50%) and conferred significant downstream-processing benefits as the *Nannochloropsis* biomass were rendered more susceptible to mechanical cell disruption. As part of their acclimation to dark anoxia, the cells upregulated various enzymes associated with heat stress, glycolysis and fermentation, as well as some proteolytic and lipolytic enzymes. The activities of lipolytic enzymes in the first 24 h of the incubation, however, appeared to be limited, with less than 10% of free fatty acid content on total lipids.

Following sustained exposure to dark anoxic conditions (24–48 h), the cells deplete the readily available carbohydrate reserves fuelling their fermentative energy generation. This resulted in significant loss of cell viability (approximately > 60% of available cells), the onset of uncontrolled intracellular degradation and the formation of free fatty acids, peroxidised lipids, and the release of proteolytic end-products from the cells. By this stage, the cessation of fermentation meant that the cells did not experience any additional cell-wall thinning. Instead, dramatic increases in proteolytic and lipolytic activities were observed, with significant protein leakage taking place from the biomass and almost 25% of free fatty acids formed based on the total lipids.

### Practical implications and outlook

Dark anoxic incubation is a low-cost method to weaken the cell walls of *Nannochloropsis* to improve the efficiency of mechanical cell rupture, and facilitate the recovery of lipid or protein products [7]. However, it was seen here to affect the integrity of the cell proteins and lipids. This section discusses the practical implications of these findings and possible future research areas that would help widen the understanding of the process.

The weakening of cell walls was found to be due to the degradation of cellulose during anoxic metabolism in *Nannochloropsis* cells [8]. However, the results here show that the cell weakening process is intertwined with the degradation of the proteins into peptides and lipids into free fatty acids and lipid peroxides. The presence of high amounts of FFAs can be detrimental for both biodiesel production [81] and food applications [21] due to the issues associated with downstream processing, and off-flavours, respectively. Other aspects of lipid chemistry are also of practical importance, warranting an in-depth analysis to elucidate the effects on the overall lipid profile in *Nannochloropsis*. Production of wax esters has been observed previously in *Euglina gracili*s in response to anoxic fermentation [69], and it is hypothesised that the absence of ethanol as a fermentative end-product in *Nannochloropsis* could be due to reactions with TAG that lead to ester formation [22]. Therefore, in addition to looking at the FFA and lipid peroxidation, an in-depth characterization of the lipid profile will provide a better understanding on how dark anoxic incubation affects the overall quality of the lipids.

When considering the progression of lipid degradation during dark anoxic incubation, it was seen that the FFA content increased most significantly after about 24 h. The late yet dramatic increase in FFA can be related to the loss of cellular integrity as a result of inadequate energy production and the inability of the cells to actively metabolize and control the activity of lipases and other catabolic enzymes [82]. Therefore, the process of incubation will need to be optimized such that it maximizes the degree of cell weakening while minimizing the degradation of the biomacromolecules to avoid the detrimental effects. As the degradation of cellulose appears to be an active pre-cursor of uncontrolled cellular degradation, it may be possible to find a more favourable incubation temperature and time combination that achieves cell weakening with minimal lipid hydrolysis. Since the results from our previous studies have demonstrated that the majority of cell-wall thinning in *Nannochloropsis* takes place within the first 24 h of dark-anoxia [8, 15], extending the incubation beyond this period can be concluded to confer no further downstream benefit. As well as valuable lipids, microalgae are rich in pigments containing antioxidative properties. In addition to the effect on the lipids, the heat stress and potential changes in the cytosolic pH could have a negative impact on these pigments and their antioxidative properties. Halim, et al. [8] found the chlorophyll pigments to degrade to pheophytin during incubation, despite the observed increase in the enzymes in the chlorophyll biosynthetic pathway. Therefore, it is worthwhile looking at the effect on the pigment profile, the total antioxidative and other bioactive properties of microalgae cells following dark anoxic incubation.

With respect to the protein content, it would be useful to determine the abundance of free amino acids during dark anoxic incubation to confirm the effects of AAT and ALAT, leading to an increase in alanine and a reduction in aspartate proposed in this study. Also, although the proteins can be considered to be ‘degraded’ into peptides, it would be worth characterising these peptides and to look for potential bioactive properties which could be of practical interest for nutritional applications. Further, given lipid oxidation occurs during dark anoxic incubation, it would be worth conducting future investigations into the extent that protein oxidation also occurs.

## Conclusions

The current study investigated the effects of dark anoxic incubation on the proteins and lipids of *Nannochloropsis* sp., including an in-depth proteomic analysis to gain a mechanistic understanding of the cellular responses. Dark anoxic incubation induced stress conditions that lead to the degradation of lipids and proteins in *Nannochloropsis* sp. over prolonged time periods. The cells altered their metabolism in response to the stress conditions of incubation, in particular the elevated temperature and dark anoxic conditions. In the absence of light and oxygen, glycolytic and fermentative pathways were used to produce energy and to maintain the redox balance of the cells during incubation. The key glycolytic and fermentative pathways used by *Nannochloropsis* cells to survive dark anoxic incubation are identified from the proteomic data obtained, and previously reported end-product analysis. Stress conditions led to the overexpression of proteins with oxidoreductase activity, gene expression, some enzymes in the glycolytic and fermentative pathways, chlorophyll biosynthesis and violaxanthin cycle, suggesting these processes were affected during incubation. Among the proteins showing higher activity, of more interest were a phospholipase, proteases and cellulases that were responsible for the lipid, protein and cellulose degradations respectively. As the incubation time increased, the energy produced through the glycolytic and fermentative pathways likely becomes inadequate to maintain the cellular integrity and to continue the metabolic processes. This culminated in the break-down of cellular integrity and the uncontrolled degradation of protein and lipids. Considering the aspects of cell weakening and the quality of microalgal products, this study emphasizes the importance of optimising the incubation time, in order to avoid or minimise the unnecessary degradation of cellular components, while still achieving the effect of cell weakening. In addition, the proposed pathways and the identified enzymes will benefit further research on *Nannochloropsis* and dark anoxic fermentation on microalgae species.

## Supplementary Information

Below is the link to the electronic supplementary material.

Supplementary file1 (DOCX 239 KB)

Supplementary file2 (XLSX 2883 KB)

## Data Availability

Data is provided within the manuscript or supplementary information files.
