# Supplementary material for: Metabolic changes and biochemical degradation during dark anoxic incubation of Nannochloropsis: implications for low-energy microalgal cell rupture
Source: Bioprocess Biosyst Eng. 2025 Jun 9;48(8):1399–420. doi: 10.1007/s00449-025-03185-7 (PMC12234632; doi:10.1007/s00449-025-03185-7)
Supplement: Supplementary file 1 — Supplementary file1 (DOCX 239 KB) [file 449_2025_3185_MOESM1_ESM.docx]

**Metabolic changes and biochemical degradation during dark anoxic incubation of *Nannochloropsis*: implications for low-energy microalgal cell rupture**

Bhagya Yatipanthalawa¹, Esther Mienis², Ronald Halim¹^,^³, Imogen Foubert², Muthupandian Ashokkumar⁴, Peter J. Scales¹, Gregory J.O. Martin¹*

¹ Algal Processing Group, Department of Chemical Engineering, The University of Melbourne, Parkville, Victoria 3010, Australia

² Research Unit Food & Lipids, KU Leuven Kulak, Department of Microbial and Molecular Systems (M^2^S), E. Sabbelaan 53, 8500 Kortrijk, Belgium

³ School of Biosystems and Food Engineering, University College Dublin, Belfield, Dublin 4, Ireland

⁴ Sonochemistry Group, School of Chemistry, The University of Melbourne, Parkville, Melbourne, Victoria 3010, Australia

0000-0001-7332-8371 (B. Yatipanthalawa), 0000-0002-0183-538X (E. Mienis), 0000-0002-7728-8888 (R. Halim), 0000-0001-8735-8926 (I. Foubert), 0000-0002-8442-1499 (M. Ashokkumar), 0000-0002-8442-1499 (P. Scales), 0000-0002-7099-7913 (G.Martin)

*Corresponding author: gjmartin@unimelb.edu.au Tel: +6183446613


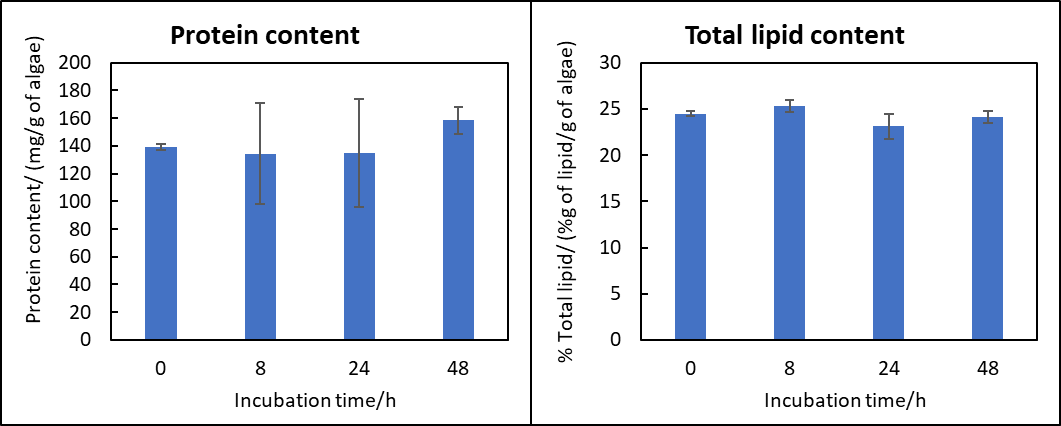


**Fig. S1** Total protein and total lipid content of *Nannochloropsis* sp.

Table S1: Proteins identified to be differentially expressed during incubation (p<0.05). Grey shading represents significantly upregulated proteins while the unshaded proteins were significantly downregulated. Protein fold increase shows the difference of the abundance of the proteins at a specific incubation time relative to that in the control, unincubated sample.

| **Majority protein IDs** | **Protein name** | **Protein fold increase compared with the non-incubated cells** | | |
| --- | --- | --- | --- | --- |
|  |  | **8h** | **24h** | **48h** |
| **Lipid metabolism** | | | | |
| A0A4D9CUP3 | PLA2c domain-containing protein | 41.10 | 43.18 | 47.41 |
| A0A4D9CUK9 | [Acyl-carrier-protein] S-malonyltransferase (EC 2.3.1.39) | 2.21 | 1.83 | 3.18 |
| A0A4D9CYN6 | Udp-sulfoquinovose synthase | 1.56 | 1.70 | 1.78 |
| **Energy/carbohydrate metabolism** | | | | |
| W7TQI2 | Endo-1,3(4)-beta-glucanase (EC 3.2.1.6) | 2.46 | 1.38 | 2.36 |
| A0A4D9D2P9 | Alpha-glucosidase ii | 1.88 | 2.77 | 3.36 |
| A0A4D9D252 | Fructokinase | 1.28 | 2.96 | 2.68 |
| W7U0D4 | Succinyl-CoA synthetase subunit alpha | 1.22 | 1.82 | 1.51 |
| A0A4D9CXW9 | Acetyl-coenzyme A synthetase (EC 6.2.1.1) | 2.09 | 2.54 | 2.95 |
| A0A4D9D596 | Dihydrolipoyllysine-residue succinyltransferase (EC 2.3.1.61) | 2.32 | 2.96 | 3.58 |
| A0A4D9CQ74 | Ferredoxin--NADP(+) reductase (EC 1.18.1.2) | 1.08 | 1.13 | 1.40 |
| A0A4D9CX97 | Dihydrolipoyl dehydrogenase (EC 1.8.1.4) | 0.75 | 0.64 | 0.49 |
| A0A4D9D2R7 | Exo-beta--glucanase | 0.45 | 0.25 | 0.34 |
| W7U1X5 | Transaldolase (EC 2.2.1.2) | 0.33 | 0.78 | 0.78 |
| A0A4D9DBL2 | Isovaleryl-dehydrogenase | 0.38 | 0.73 | 0.56 |
| A0A4D9D7Z1 | UDPglucose pyrophosphorylase | 0.70 | 0.52 | 0.90 |
| A0A4D9DC52 | Rhamnose biosynthetic enzyme expressed | 0.33 | 0.44 | 0.47 |
| **Proteases and protein metabolism** | | | | |
| A0A4D9CPY1 | 26s protease regulatory subunit 8 | 13.11 | 11.14 | 13.31 |
| A0A4D9D6A4 | ATP-dependent Clp protease proteolytic subunit | 0.79 | 0.98 | 3.60 |
| T1RI58 | ATP-dependent zinc metalloprotease FtsH (EC 3.4.24.-) | 1.17 | 1.15 | 1.56 |
| A0A4D9D8Q8 | Ubiquitin-activating enzyme e1 | 0.94 | 1.39 | 2.14 |
| A0A4D9D849 | Ubiquitin-conjugating enzyme catalytic domain protein | 2.55 | 2.99 | 6.10 |
| W7TFQ1 | Mitochondrial-processing peptidase subunit beta | 1.51 | 1.79 | 1.49 |
| A0A4D9CWF8 | Carboxyl-terminal processing protease | 0.54 | 0.35 | 0.16 |
| A0A4D9D808 | 26S proteasome regulatory subunit RPN11 | 0.80 | 0.47 | 0.58 |
| **Gene expression** | | | | |
| K9ZV80 | 30S ribosomal protein S10, chloroplastic | 88.63 | 112.40 | 137.40 |
| K9ZV97 | 30S ribosomal protein S18, chloroplastic | 2.82 | 3.93 | 1.98 |
| A0A4D9CZW2 | 50S ribosomal protein L5, chloroplastic | 2.46 | 1.87 | 3.08 |
| A0A4D9D8D6 | Uncharacterized protein | 4.10 | 2.39 | 4.13 |
| A0A4D9D6Q8 | 30s ribosomal protein s1 | 0.87 | 0.80 | 1.26 |
| W7THS5 | Peptidyl-prolyl cis-trans isomerase (PPIase) (EC 5.2.1.8) | 2.18 | 2.26 | 3.88 |
| W7TD66 | Peptidyl-prolyl cis-trans isomerase (PPIase) (EC 5.2.1.8) | 1.41 | 1.36 | 1.86 |
| A0A4D9D029 | Rna binding protein | 2.30 | 2.81 | 0.67 |
| A0A4D9D910 | Rna binding s1 domain protein | 1.73 | 1.68 | 1.68 |
| W7UA52 | Histone h3 | 2.32 | 4.41 | 4.77 |
| W7TJJ6 | Proliferating cell nuclear antigen | 1.43 | 3.73 | 2.76 |
| A0A4D9CVG6 | Elongation factor ef-3 | 1.23 | 1.29 | 10.31 |
| W7TRP0 | Eukaryotic initiation factor 4a | 1.12 | 0.92 | 1.76 |
| W7TD18 | DNA ligase (EC 6.5.1.1), Ribulose-phosphate 3-epimerase (EC 5.1.3.1) | 1.12 | 1.70 | 0.99 |
| A0A4D9CXK5 | U1 small nuclear ribonucleoprotein a | 4.13 | 9.89 | 7.11 |
| A0A4D9CYW1 | Adenylosuccinate lyase (ASL) (EC 4.3.2.2) (Adenylosuccinase) | 5.28 | 7.84 | 4.74 |
| A0A4D9D2A5 | GrpE nucleotide exchange factor | 1.48 | 1.23 | 2.17 |
| A0A4D9CVP5 | Chaperonin cpn60 tcp-1 | 0.89 | 1.20 | 1.85 |
| W7TPW8 | Rubisco expression protein | 1.65 | 1.56 | 1.58 |
| A0A4D9D9K7 | Prolyl-tRNA synthetase (EC 6.1.1.15) | 0.76 | 1.35 | 2.04 |
| A0A4D9DB93 | Asparagine--tRNA ligase (EC 6.1.1.22) | 1.74 | 2.15 | 4.94 |
| A0A4D9D1E6 | Uncharacterized protein | 0.36 | 0.52 | 0.45 |
| A0A4D9DC93 | Peptidyl-prolyl cis-trans isomerase | 0.45 | 0.38 | 0.42 |
| A0A4D9DAP6 | Peptidylprolyl isomerase (EC 5.2.1.8) | 0.63 | 0.47 | 0.32 |
| W7TWV9 | Peptidylprolyl isomerase (EC 5.2.1.8) | 0.89 | 0.50 | 0.14 |
| A0A4D9D874 | Peptidylprolyl isomerase (EC 5.2.1.8) | 0.19 | 0.17 | 0.34 |
| **Amino acid biosynthesis** | | | | |
| A0A4D9CQT5 | Acetohydroxy-acid reductoisomerase | 0.94 | 1.01 | 1.39 |
| W7TX01 | Pyrroline-5-carboxylate reductase (EC 1.5.1.2) | 1.01 | 1.28 | 1.35 |
| A0A4D9CME1 | Aspartate aminotransferase | 1.65 | 2.34 | 1.80 |
| A0A4D9CVJ1 | Branched-chain amino acid aminotransferase | 0.37 | 0.27 | 0.42 |
| A0A4D9CYV5 | Cysteine synthase | 0.46 | 0.37 | 0.39 |
| **Oxidoreductases and stress induced proteins** | | | | |
| A0A4D9D3X6 | Betaine aldehyde dehydrogenase | 1.30 | 3.52 | 4.64 |
| A0A4D9D429 | Short-chain dehydrogenase reductase sdr | 5.22 | 5.93 | 6.23 |
| W7UAI2 | PKS_ER domain-containing protein, Cinnamyl alcohol dehydrogenase | 2.28 | 4.32 | 4.74 |
| A0A4D9D7K1 | Heat shock protein | 1.96 | 1.77 | 2.28 |
| A0A4D9CWW7 | Stress-inducible protein sti1 | 1.62 | 2.45 | 2.45 |
| A0A4D9D323 | Obtusifoliol 14-alpha demethylase | 1.37 | 1.32 | 2.32 |
| A0A4D9CXV0 | Hypersensitive-induced response protein,PHB domain-containing protein | 2.20 | 2.81 | 1.96 |
| A0A4D9D7Y8 | Glutathione s-transferase | 1.82 | 2.83 | 3.03 |
| A0A4D9CUC9 | Nucleoredoxin | 0.96 | 1.25 | 1.65 |
| A0A4D9D7N4 | Arogenate dehydrogenase | 1.30 | 2.44 | 2.03 |
| A0A4D9CYT4 | Short-chain dehydrogenase | 0.47 | 0.23 | 0.14 |
| A0A4D9D107 | VKc domain-containing protein | 0.52 | 0.20 | 0.19 |
| A0A4D9D5I9 | Cytochrome c peroxidase | 0.37 | 0.38 | 0.39 |
| W7TH17 | Glutathione peroxidase | 0.48 | 0.30 | 0.12 |
| A0A4D9DC12 | Glutathione reductase (EC 1.8.1.7) | 0.36 | 0.27 | 0.22 |
| **Signalling proteins and transport** | | | | |
| A0A4D9DC09 | PDZ domain protein | 5.19 | 6.91 | 10.80 |
| A0A4D9CQ73 | MFS domain-containing protein | 1.55 | 4.21 | 6.82 |
| A0A4D9CY02 | metal ion transmembrane transporter activity [GO:0046873] | 1.90 | 2.89 | 1.07 |
| A0A4D9D9I1 | Mitochondrial carrier | 1.59 | 2.85 | 3.44 |
| A0A4D9CTY7 | Amino acid transporter, transmembrane | 3.94 | 3.16 | 4.40 |
| A0A4D9D428 | Protein translocase subunit SecA | 1.06 | 1.11 | 1.70 |
| A0A4D9CNB5 | Myosin light chain kinase, Protein kinase domain-containing protein | 0.92 | 1.68 | 1.97 |
| **Photosynthesis and chlorophyll biosynthesis** | | | | |
| A0A4D9DCN1 | Geranylgeranyl reductase (EC 1.3.1.83) | 1.02 | 1.06 | 1.42 |
| T1RGM9 | Magnesium chelatase (EC 6.6.1.1) | 1.77 | 1.92 | 2.30 |
| T1RH13 | Photosystem I reaction center subunit VIII (PSI-I) | 3.15 | 1.54 | 4.22 |
| A0A4D9DHT9 | Glutamyl-tRNA synthetase (EC 6.1.1.17) | 1.23 | 1.60 | 2.87 |
| W7TX20 | Light-harvesting protein | 0.75 | 0.93 | 0.67 |
| W7T8I0 | Light-harvesting protein | 0.56 | 0.38 | 0.56 |
| W7TCZ6 | Mg chelatase subunit | 0.13 | 0.29 | 0.78 |
| K9ZXJ9 | Cytochrome c-550 (Cytochrome c550) | 0.92 | 0.56 | 0.21 |
| **Violaxanthin cycle** | | | | |
| A0A4D9DAF3 | Violaxanthin de-epoxidase-related protein | 4.86 | 4.47 | 5.16 |
| A0A4D9CSI8 | Zeaxanthin epoxidase | 0.99 | 1.36 | 1.67 |
| **Metabolism** | | | | |
| A0A4D9CXT2 | Rnase l inhibitor-like protein | 2.16 | 1.77 | 4.67 |
| A0A4D9D739 | Putative cation-transporting atpase 13a1 | 20.70 | 39.09 | 22.44 |
| W7TSQ4 | S-adenosylmethionine synthase (EC 2.5.1.6) | 0.84 | 0.57 | 0.48 |
| K9ZWY0 | AAA family ATPase (Putative AAA domain-containing protein Ycf46 | 0.36 | 0.43 | 0.56 |
| A0A4D9CUN8 | Pab-dependent poly-specific ribonuclease subunit 3 | 0.24 | 0.05 | 0.14 |
| A0A4D9DDQ5 | Phosphatidylinositide phosphatase sac1 | 0.47 | 0.49 | 0.72 |
| **Others** | | | | |
| A0A4D9CQD3 | AMP-binding domain-containing protein | 2.39 | 2.65 | 2.89 |
| A0A4D9DBW7 | Proton-translocating NAD(P)(+) transhydrogenase (EC 7.1.1.1) | 3.86 | 3.40 | 4.13 |
| W7TGL9 | Receptor expression-enhancing protein 6 | 1.01 | 1.90 | 1.92 |
| W7TSH6 | Tautomerase | 2.73 | 3.35 | 2.57 |
| W7TFK4 | Tubulin alpha chain | 2.39 | 4.11 | 3.34 |
| A0A4D9DGD2 | Asp_Arg_Hydrox domain-containing protein | 4.98 | 13.71 | 15.46 |
| A0A4D9D9S4 | Methyltransf_11 domain-containing protein | 0.15 | 0.09 | 0.10 |
| W7T5W5 | Methyltransf_11 domain-containing protein | 0.46 | 0.79 | 1.03 |
| W7TIQ3 | Protein-l-isoaspartate (D-aspartate) o- methyltransferase | 0.55 | 0.39 | 0.30 |
| A0A4D9DAM0 | ATP-dependent (S)-NAD(P)H-hydrate dehydratase (EC 4.2.1.93) (ATP-dependent NAD(P)HX dehydratase) | 0.52 | 0.58 | 0.28 |
| A0A4D9D2R3 | Nad-dependent epimerase dehydratase | 0.55 | 0.28 | 0.27 |
| A0A4D9DAA6 | Tpr repeat protein | 0.19 | 0.05 | 0.06 |
| A0A4D9D997 | Transmembrane protein, DUF1279 domain-containing protein | 0.62 | 0.55 | 0.63 |
| A0A4D9CY79 | VOC domain-containing protein | 0.88 | 1.07 | 0.59 |
| A0A4D9D9E7 | Ycii-like protein | 0.28 | 0.34 | 0.40 |
| A0A4D9DCC5 | ZnF_CDGSH domain-containing protein | 0.77 | 0.67 | 0.15 |


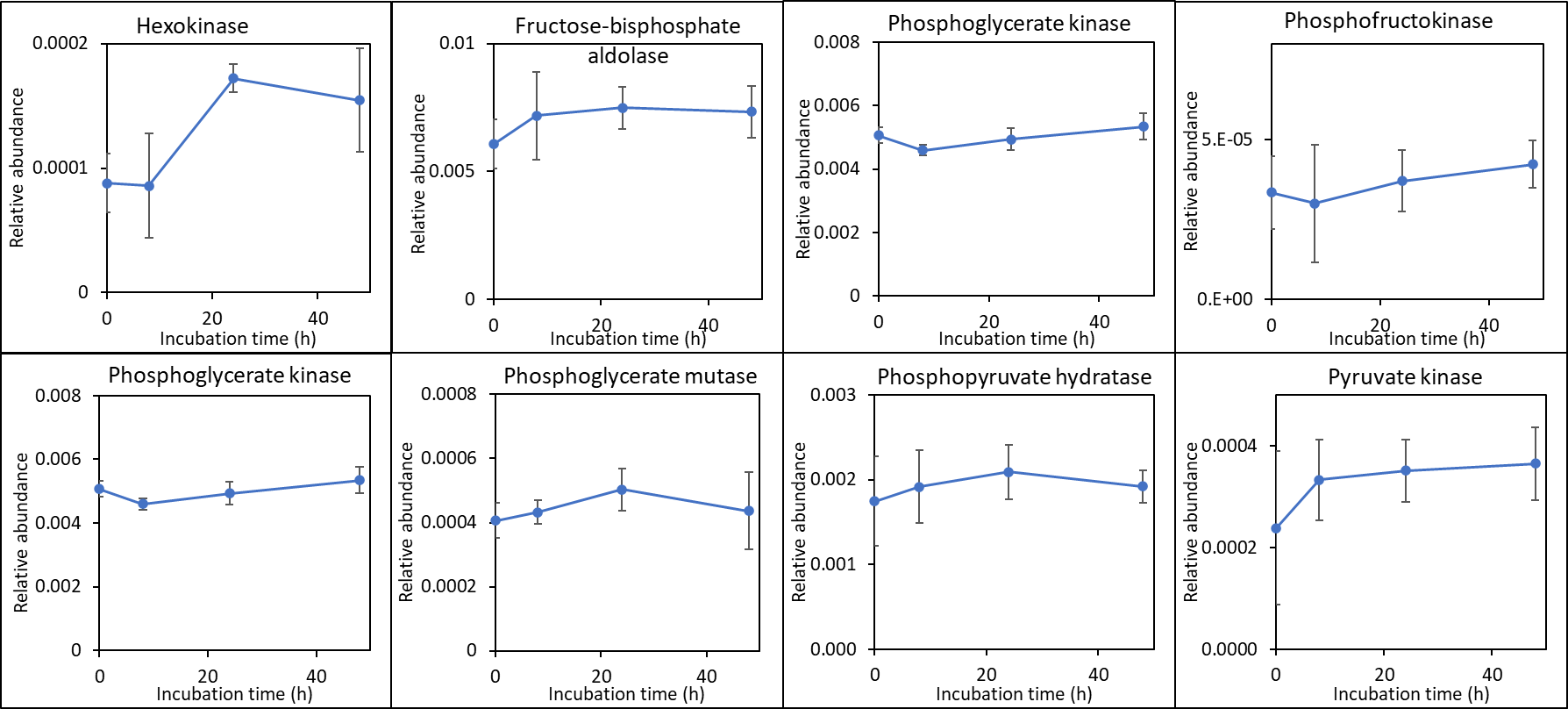


**Fig. S2** The average sum of the relative abundance of proteins involved in the glycolytic pathway.


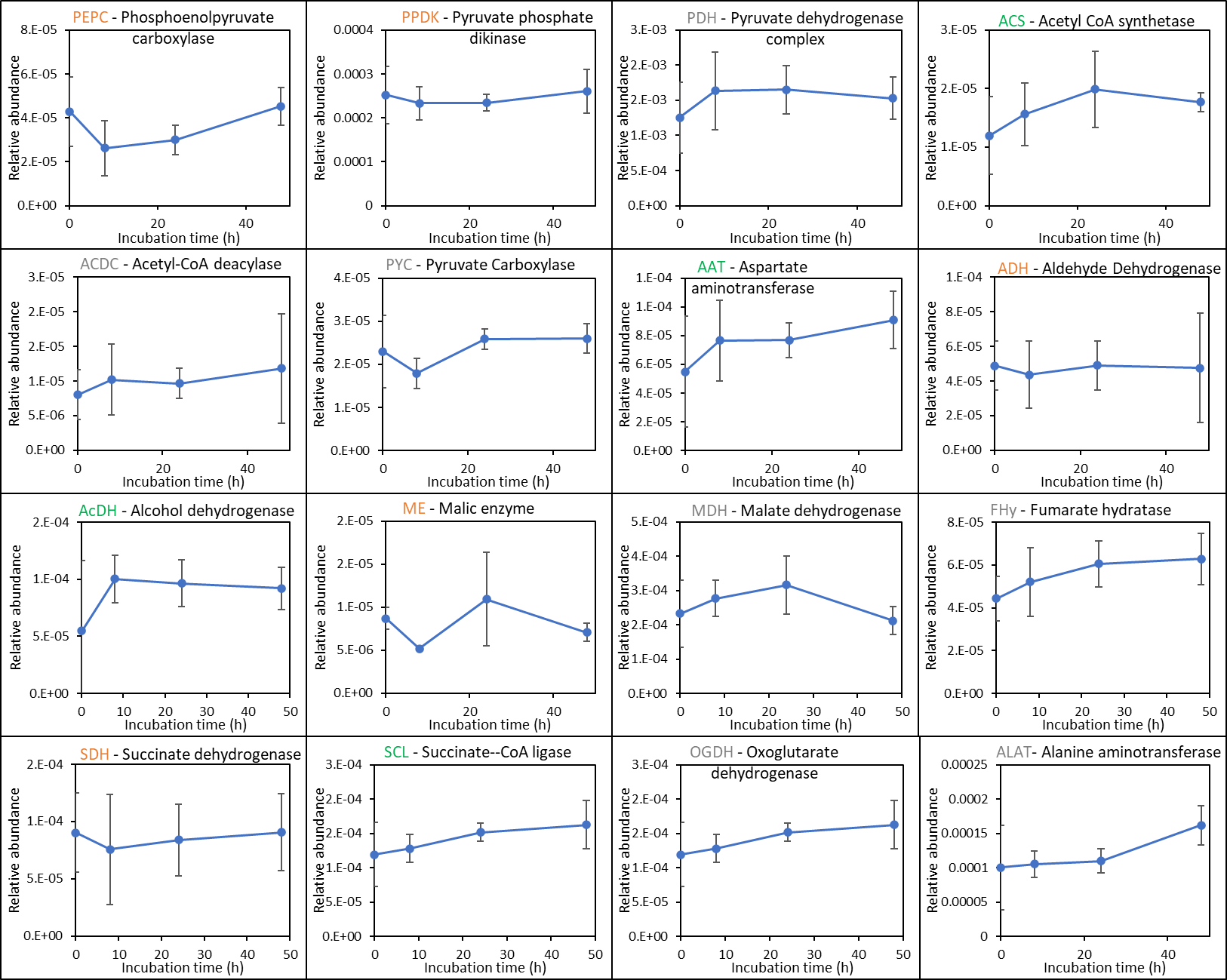


**Fig. S3** The average sum of the relative abundance of proteins involved in the fermentative pathways.
